# Adaptive and optimized COVID-19 vaccination strategies across geographical regions and age groups

**DOI:** 10.1371/journal.pcbi.1009974

**Published:** 2022-04-07

**Authors:** Jeta Molla, Alejandro Ponce de León Chávez, Takayuki Hiraoka, Tapio Ala-Nissila, Mikko Kivelä, Lasse Leskelä

**Affiliations:** 1 Department of Applied Physics, Aalto University, Espoo, Finland; 2 Department of Mathematics and Systems Analysis, Aalto University, Espoo, Finland; 3 Department of Computer Science, Aalto University, Espoo, Finland; 4 Quantum Technology Finland Center of Excellence and Department of Applied Physics, Aalto University, Espoo, Finland; 5 Interdisciplinary Centre for Mathematical Modelling and Department of Mathematical Sciences, Loughborough University, Loughborough, United Kingdom; Fundação Getúlio Vargas: Fundacao Getulio Vargas, BRAZIL

## Abstract

We evaluate the efficiency of various heuristic strategies for allocating vaccines against COVID-19 and compare them to strategies found using optimal control theory. Our approach is based on a mathematical model which tracks the spread of disease among different age groups and across different geographical regions, and we introduce a method to combine age-specific contact data to geographical movement data. As a case study, we model the epidemic in the population of mainland Finland utilizing mobility data from a major telecom operator. Our approach allows to determine which geographical regions and age groups should be targeted first in order to minimize the number of deaths. In the scenarios that we test, we find that distributing vaccines demographically and in an age-descending order is not optimal for minimizing deaths and the burden of disease. Instead, more lives could be saved by using strategies which emphasize high-incidence regions and distribute vaccines in parallel to multiple age groups. The level of emphasis that high-incidence regions should be given depends on the overall transmission rate in the population. This observation highlights the importance of updating the vaccination strategy when the effective reproduction number changes due to the general contact patterns changing and new virus variants entering.

## Author summary

The COVID-19 vaccines are now available worldwide and many countries follow the practice of distributing them heuristically e.g. in age-descending order and demographically. Here we evaluate the effectiveness of such strategies by comparing them with optimized ones from an age and spatially-structured mathematical model of COVID-19 transmission. We find that vaccinating multiple age groups simultaneously and targeting regions with the the highest incidence can save more lives than heuristic strategies. Our work also reveals the importance of assessing the vaccination strategy at different stages of the epidemic.

This is a *PLOS Computational Biology* Methods paper.

## Introduction

With reports of around three million deaths and 160 million cases worldwide [1], the COVID-19 pandemic has caused a global public health crisis with far-reaching consequences to the economy and lives of people. Vaccines promise a way out of this situation, but due to limited supply and finite rate of vaccination they are not immediately effective in eradicating the epidemic. Health officials and governments around the world are thus faced with decisions on which order to vaccinate the population. This can be a matter of life and death to a large number of people and determine the speed at which we steer out of the crisis. The problem at hand is complicated by different mortality rates and activity levels in different age groups, localised incidence rates, and mobility patterns between regions, making it difficult to find an optimal solution on how to vaccinate using heuristic arguments. Given the scope of the crisis, even a small change in the relative efficiency of a strategy can have a large impact at the absolute scale in terms of saving lives. Therefore, critical evaluation on different vaccination strategies is imperative.

Several studies have previously explored the effectiveness of different age-structured vaccination strategies against the COVID-19 [2–8]. Most of them agree that for minimizing cumulative incidence, i.e., the number of individuals who experience infection by the end of the epidemic, it is optimal to give priority to younger generations, as their higher activity accounts for a large part of the transmission. However, if the minimization of deaths and hospitalizations is targeted, it is often preferable to allocate vaccines first to the elderly who have a higher risk of severe illness and death. The set of strategies considered in the aforementioned studies is limited to sequential vaccinations of different age groups. They do not take into account parallel vaccination across age groups nor other factors such as the mobility and contact patterns of individuals. Further, suitable geographical distribution of vaccines is important especially when prevalence is inhomogeneously distributed across different geographical regions. Bertsimas et al. [9] and Grauer et al. [10] have shown that allocating vaccines to regions with high incidence can reduce the number of deaths compared to the strategy of distributing vaccines demographically. Further, Lemaitre et al. [11] have studied optimal spatial allocation of COVID-19 vaccines via an optimal control framework taking into account the mobility network and the spatial heterogeneities. Ideally, all aforementioned factors should be optimized simultaneously, but once we start to take into account such parallel and region-based prioritization strategies, the space of possible strategies becomes so large that a brute-force search for an optimal strategy is no longer feasible; hence we need an efficient algorithm for finding a strategy that optimizes the given objective function.

To this end, we here construct an epidemic model that takes into account the various factors mentioned above. We use the model to study the effectiveness of different vaccination strategies by nonlinear optimization methods. The epidemic progression is described by a deterministic compartmental model adapted to COVID-19. As a case study, we adjust the model parameters to the recent epidemic situation on mainland Finland. Based on census data, age-structured contact patterns, and mobility patterns from a mobile phone operator, we infer contact patterns between individuals in different regions and age groups. Based on the available data of reported cases and vaccination counts, the performance of several vaccination strategies that are implemented or considered by health authorities is evaluated by means of a nonlinear programming framework. This framework allows us to optimize age-based and region-based vaccination schedules. As our main result, we find that the heuristic strategy of vaccinating the high-risk groups serially and distributing vaccines uniformly based on the local population density may not be optimal in minimizing deaths and mitigating the disease burden. Instead, better results can be obtained by parallel vaccination of different age groups and geographically targeted distribution of vaccines in a way that adapts to the ongoing incidence over time and takes into account demographic and behavioral differences across different regions. This calls for re-evaluation of the details of any chosen vaccination strategy during the course of vaccinating the population.

## Methods

The level of detail in modelling epidemic spreading dynamics depends both on the questions that need to be answered and the availability of relevant data. One of the characteristic features of the COVID-19 epidemic is the large heterogeneity in mortality across different age groups. For evaluating vaccination strategies, we also need to include the initial state of the epidemic at a given time, the arrival rate of new vaccine doses and their efficacy, and contact patterns between individuals of different ages for transmission rates. The final complication comes from geographic heterogeneity which requires local population densities and accurate mobility data between different regions.

### Region- and age-based epidemiological model

We introduce a deterministic compartmental model of COVID-19 transmission and vaccination which takes into account both heterogeneities across age groups and mobility across geographical regions. We assume that new vaccine doses arrive at a constant rate and all types of vaccines have equal efficacy. We consider an extension of the all-or-nothing model [2, 12] in order to take into account individual variations in immunity. To this end some vaccinated individuals develop full immunity while others have only partial protection against transmission and severe illness after receiving the first dose. The proportion of individuals accepting to be vaccinated is assumed to be constant across the population.

The population of a country is modelled as a closed system of *N* individuals, divided into regions *k* = 1, …, *K* and age groups *g* = 1, …, *G*. An individual resident in region *k* in age group *g* is called a *kg*-individual, and the number of such individuals is denoted by *N*_*kg*_. In what follows, *g*, *h* always refer to age, and *k*, *ℓ*, *m* to regions. The population size of region *k* is denoted by *N*_*k*_ = ∑_*g*_ *N*_*kg*_, and the size of age group *g* by *N*_*g*_ = ∑_*k*_ *N*_*kg*_.

The population in each stratum is divided into 16 time-dependent epidemiological compartments described in Table 1.

**Table 1:** Epidemiological compartments. There are *KG* copies of each compartment, denoted $S_{kg}^{u},S_{kg}^{v},\ldots ,V_{kg}$ for regions *k* = 1, …, *K* and age groups *g* = 1, …, *G*.

Symbol	Description
*S* ^*u*^	Susceptible, unvaccinated
*S* ^*v*^	Susceptible, invited for vaccination
*S* ^*x*^	Susceptible, unable or unwilling to be vaccinated
*S* ^*p*^	Susceptible, developed weak immunity after vaccination
*E*	Infected but not yet infectious
*E* ^*v*^	Vaccinated infected but not yet infectious
*I*	Infected and infectious
*I* ^*v*^	Vaccinated infected and infectious
*Q* ^0^	Quarantined at home, mild disease
*Q* ^1^	Quarantined at home, severe disease
*H* ^*w*^	Hospitalized, in general ward
*H* ^*c*^	Hospitalized, in critical care
*H* ^*r*^	Hospitalized, in recovery ward
*D*	Deceased
*R*	Recovered with full immunity
*V*	Vaccinated with full immunity

**Fig 1:**
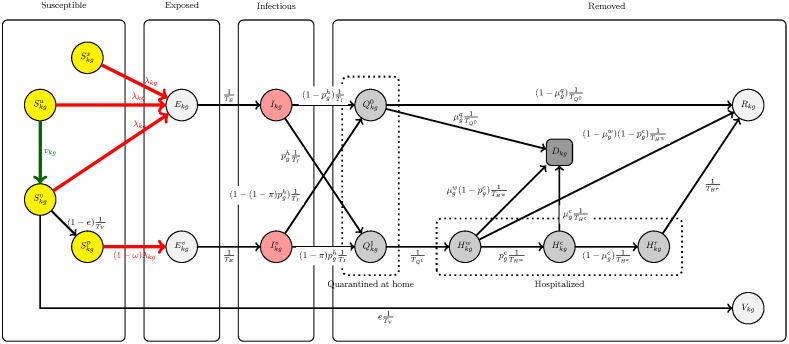
Disease transmission dynamics. Each node in the diagram corresponds to one differential equation with the time derivative of the associated variable on the left side, the values of the source nodes of incident arrows on the right side, each incoming arrow equipped with a plus sign, and each outgoing arrow equipped with a minus sign.

The dynamics of the disease is modelled using a deterministic nonlinear system of 16*KG* ordinary differential equations with structure shown in Fig 1. We treat the variables as expectation values, so they may take non-integer values. This leads to a system where susceptible compartments evolve according to

$$\begin{array}{c} \begin{array}{cc} \frac{d}{dt}S_{kg}^{x} & \;=\;-\lambda_{kg}S_{kg}^{x},\\ \frac{d}{dt}S_{kg}^{u} & \;=\;-\lambda_{kg}S_{kg}^{u}-v_{kg}S_{kg}^{u},\\ \frac{d}{dt}S_{kg}^{v} & \;=\;v_{kg}S_{kg}^{u}-\lambda_{kg}S_{kg}^{v}-\frac{1}{T_{V}}S_{kg}^{v},\\ \frac{d}{dt}S_{kg}^{p} & \;=\;\left(1-e\right)\frac{1}{T_{V}}S_{kg}^{v}-\left(1-\omega \right)\lambda_{kg}S_{kg}^{p}, \end{array} \end{array}$$ (1)

infected but noninfectious compartments according to

$$\begin{array}{c} \begin{array}{cc} \frac{d}{dt}E_{kg} & \;=\;\lambda_{kg}\left(S_{kg}^{x}+S_{kg}^{u}+S_{kg}^{v}\right)-\frac{1}{T_{E}}E_{kg},\\ \frac{d}{dt}E_{kg}^{v} & \;=\;\left(1-\omega \right)\lambda_{kg}S_{kg}^{p}-\frac{1}{T_{E}}E_{kg}^{v}, \end{array} \end{array}$$ (2)

infectious compartments according to

$$\begin{array}{c} \begin{array}{cc} \frac{d}{dt}I_{kg} & \;=\;\frac{1}{T_{E}}E_{kg}-\frac{1}{T_{I}}I_{kg},\\ \frac{d}{dt}I_{kg}^{v} & \;=\;\frac{1}{T_{E}}E_{kg}^{v}-\frac{1}{T_{I}}I_{kg}^{v} \end{array} \end{array}$$ (3)

and removed compartments according to

$$\begin{array}{c} \begin{array}{cc} \frac{d}{dt}Q_{kg}^{0} & \;=\;\left(1-p_{g}^{h}\right)\frac{1}{T_{I}}I_{kg}+\left(1-\left(1-\pi \right)p_{g}^{h}\right)\frac{1}{T_{I}}I_{kg}^{v}-\frac{1}{T_{Q^{0}}}Q_{kg}^{0},\\ \frac{d}{dt}Q_{kg}^{1} & \;=\;p_{g}^{h}\frac{1}{T_{I}}\left(I_{kg}+\left(1-\pi \right)I_{kg}^{v}\right)-\frac{1}{T_{Q^{1}}}Q_{kg}^{1},\\ \frac{d}{dt}H_{kg}^{w} & \;=\;\frac{1}{T_{Q^{1}}}Q_{kg}^{1}-\frac{1}{T_{H^{w}}}H_{kg}^{w},\\ \frac{d}{dt}H_{kg}^{c} & \;=\;p_{g}^{c}\frac{1}{T_{H^{w}}}H_{kg}^{w}-\frac{1}{T_{H^{c}}}H_{kg}^{c},\\ \frac{d}{dt}H_{kg}^{r} & \;=\;\left(1-\mu_{g}^{c}\right)\frac{1}{T_{H^{c}}}H_{kg}^{c}-\frac{1}{T_{H^{r}}}H_{kg}^{r},\\ \frac{d}{dt}R_{kg} & \;=\;\left(1-\mu_{g}^{q}\right)\frac{1}{T_{Q^{0}}}Q_{kg}^{0}+\left(1-\mu_{g}^{w}\right)\left(1-p_{g}^{c}\right)\frac{1}{T_{H^{w}}}H_{kg}^{w}+\frac{1}{T_{H^{r}}}H_{kg}^{r},\\ \frac{d}{dt}D_{kg} & \;=\;\mu_{g}^{q}\frac{1}{T_{Q^{0}}}Q_{kg}^{0}+\mu_{g}^{w}\left(1-p_{g}^{c}\right)\frac{1}{T_{H^{w}}}H_{kg}^{w}+\mu_{g}^{c}\frac{1}{T_{H^{c}}}H_{kg}^{c},\\ \frac{d}{dt}V_{kg} & \;=\;e\frac{1}{T_{V}}S_{kg}^{v}. \end{array} \end{array}$$ (4)

In formulae (1)–(4), the force of infection inflicted on *kg* susceptibles λ_*kg*_ = λ_*kg*_(*t*) varies over time as a function of infectious states in all strata and additional parameters. The force of infection (per capita rate of infections) inflicted on susceptible *kg* individuals equals

$$\begin{array}{c} \lambda_{kg}\left(I\right)\;=\;\beta \;\sum_{m,\ell ,h}\frac{\beta_{gh}}{{\hat{N}}_{m}}\theta_{km}\left(I_{\ell h}+\xi I_{\ell h}^{v}\right)\theta_{\ell m}, \end{array}$$ (5)

where *β* is a constant used for adjusting the overall rate of infectious contacts, (*β*_*gh*_) is a 9-by-9 mobility-adjusted age contact matrix, (*θ*_*kℓ*_) is a 5-by-5 baseline mobility, and ${\hat{N}}_{m}$ is the effective population size of region *m*. This corresponds to a model where $\beta \times \beta_{gh}/{\hat{N}}_{m}$ is the contact rate between any unordered pair of individuals present in region *m*, with one individual belonging to age group *g* and the other to age group *h*.

The per-capita rate of vaccines offered to residents of region *k* in age group *g* is a time-dependent function *v*_*kg*_ = *v*_*kg*_(*t*) obtained as a solution of a minimization problem, or defined manually. The other model parameters are constant and are listed in Table 2.

**Table 2:** Parameters for the epidemic model. The parameters here have been taken from Ref. [12] except for the vaccine efficacy *e* which depends on several factors including the vaccination type, disease variant, number of doses and time from the vaccination [13–15]. Here we set *e* following Ref. [16].

	Description	0–9	10–19	20–29	30–39	40–49	50–59	60–69	70–79	80+
*T*_*E*_	Latent period(days)	3	3	3	3	3	3	3	3	3
*T*_*I*_	Transmission period (days)	4	4	4	4	4	4	4	4	4
$T_{Q^{0}}$	Quarantine period with mild symptoms (days)	5	5	5	5	5	5	5	5	5
$T_{Q^{1}}$	Quarantine period with severe symptoms (days)	3	3	3	3	3	3	3	3	3
$T_{H^{w}}$	Hospital ward period (days)	5	5	5	5	5	5	5	5	5
$T_{H^{c}}$	Critical care period (days)	9	9	9	9	9	9	9	9	9
$T_{H^{r}}$	Post-critical care period (days)	1	1	1	1	1	1	1	1	1
*T*_*V*_	Vaccination immunity delay (days)	10	10	10	10	10	10	10	10	10
$p_{g}^{h}$	Fraction of severe cases	0	0	0.02	0.03	0.04	0.08	0.16	0.43	0.52
$p_{g}^{c}$	Fraction of critical cases among severe	0	0	0	0	0	0.01	0.03	0.05	0.01
$\mu_{g}^{q}$	Fraction of non-hospitalized that die	0	0	0	0	0	0	0	0.08	0.2
$\mu_{g}^{h}$	Fraction of hospitalized that die	0	0	0	0	0	0	0	0.2	0.4
$\mu_{g}^{c}$	Fraction of inds. in critical care that die	0.35	0.1	0.1	0.15	0.15	0.22	0.46	0.49	0.52
*e*	Vaccine efficacy to confer full immunity	0.7	0.7	0.7	0.7	0.7	0.7	0.7	0.7	0.7
*ω*	Vaccine efficacy to reduce susceptibility	0.4	0.4	0.4	0.4	0.4	0.4	0.4	0.4	0.4
*π*	Protection against severe illness	0.6	0.6	0.6	0.6	0.6	0.6	0.6	0.6	0.6
*ξ*	Reduction in transmissibility	1	1	1	1	1	1	1	1	1
*τ*	Fraction of daily activity spent in a region	0.5	0.5	0.5	0.5	0.5	0.5	0.5	0.5	0.5

In our numerical investigations, the population is stratified into 9 age groups and 5 geographical regions (Table 3), giving us total of 45 age-region strata. Per each stratum, there are 16 epidemiological compartments, including three susceptible compartments (unvaccinated, vaccinated with developing immunity, and vaccinated without developing immunity) and two tracks (mild and severe) of infected individuals. This leads to a full model with 720 age-region-compartment combinations.

**Table 3:** Population, incidence (7-day case notification rate per 100 000 individuals), and vaccine uptake (proportion of vaccinated with first dose per 100 individuals) in five regions (university hospital specific catchment areas) of mainland Finland on 18 April 2021.

Region	Population	Incidence	Vaccine uptake
HYKS	2 198 182	53.6	23.4
TYKS	869 004	39.9	26.9
TAYS	902 681	24.9	25.2
KYS	797 234	10.0	25.4
OYS	736 563	10.3	22.7
Total	5 503 664	34.7	24.4

### Mobility

Mobility of individuals is modelled using a Lagrangian approach [17] using a *K*-by-*K* probability matrix where entry *θ*_*kℓ*_ equals the fraction of time that a typical resident of region *k* spends in region *ℓ*. Then

$$\begin{array}{c} {\hat{N}}_{\ell g}\;=\;\sum_{k}N_{kg}\theta_{k\ell } \end{array}$$ (6)

equals the mean number of individuals of age group *g* present in region *ℓ*, and

$${\hat{N}}_{\ell }\;=\;\sum_{g}{\hat{N}}_{\ell g}$$

represents the mean number of individuals present in region *ℓ*.

The baseline mobility matrix representing typical mobility in Finland during normal times without pandemic is a 5-by-5 matrix with entries estimated from available data on cross-region travels as

$$\begin{array}{c} \theta_{km}\;=\;(\left(1-\tau \right)+\tau (1-\frac{\psi_{k+}}{N_{k}}))\delta_{km}+\tau \frac{\psi_{km}}{N_{k}}\left(1-\delta_{km}\right), \end{array}$$ (7)

where *ψ*_*k*+_ = ∑_*m*≠*k*_ *ψ*_*km*_, (*ψ*_*km*_) is an estimated trip matrix with *ψ*_*km*_ telling the daily number of trips that residents of region *k* make to region *m* in Table C in S1 Appendix, *N*_*k*_ is the number of residents in region *k* obtained from in Table B in S1 Appendix, and *δ*_*km*_ is the Kronecker delta. The parameter *τ* represents the fraction of daily activity time that a typical commuter spends in a remote region. In our numerical simulations we set *τ* = {0, 0.5, 1} due to lack of reliable data for estimating this factor. Eq (7) can be interpreted as the expected fraction of active day time that a resident of region *k* spends in region *m*, with *ψ*_*k*+_/*N*_*k*_ being the probability that a randomly selected resident of region *k* commutes outside the home region on a given day.

### Calibration of the overall infectious contact rate

The overall infectious contact rate parameter *β* is parameterised in terms of an effective reproduction number *R*_eff_ as follows. Denote by *K*^(*β*)^ a *KG*-by-*KG* matrix with entries

$$K_{kg,\ell h}^{\left(\beta \right)}\;=\;\beta T_{I}S_{kg}\left(0\right)M_{kg,\ell h},$$

where

$$M_{kg,\ell h}\;=\;\beta_{gh}\sum_{m}\frac{\theta_{km}\theta_{\ell m}}{{\hat{N}}_{m}},$$

and $S_{kg}\left(0\right)=S_{kg}^{u}\left(0\right)+S_{kg}^{v}\left(0\right)+S_{kg}^{x}\left(0\right)$ is the number of *kg* susceptibles at time zero. The variable $K_{kg,\ell h}^{\left(\beta \right)}$ indicates the expected number of new infections among *kg* individuals caused by an infectious *ℓh* individual who got infected at time zero. Then we set

$$\beta \;=\;\frac{R_{\text{eff}}}{\rho (K^{\left(1\right)})},$$

where *ρ*(*K*^(1)^) is the spectral radius of the matrix *K*^(1)^ = *T*_*I*_ *S*_*kg*_(0)*M*_*kg*,*ℓh*_, and *R*_eff_ is set to values 0.75, 1.0, 1.25, 1.50 in different scenarios. With this choice, the spectral radius of *K*^(*β*)^ equals *ρ*(*K*^(*β*)^) = *R*_eff_, and *R*_eff_ < 1 (resp. *R*_eff_ > 1) indicates the convergence to zero (resp. divergence) of a subsystem of differential equations

$$\begin{array}{cc} \frac{d}{dt}E_{kg} & =\;\beta S_{kg}\left(0\right)\sum_{\ell h}M_{kg,\ell h}I_{\ell h}-\frac{1}{T_{E}}E_{kg},\\ \frac{d}{dt}I_{kg} & =\;\frac{1}{T_{E}}E_{kg}-\frac{1}{T_{I}}I_{kg}, \end{array}$$

only containing the infectious compartments, linearised in a neighbourhood of a stable initial state where $S_{kg}^{u}\left(0\right),S_{kg}^{v}\left(0\right),S_{kg}^{x}\left(0\right)$ are fixed to their current states, and *E*_*kg*_ = *I*_*kg*_ = 0 for all *kg*, see [18, 19]. Hence *R*_eff_ < 1 indicates that all infectious compartments would decrease locally in time even without future vaccinations. In the special case where *S*_*kg*_(0) = *N*_*kg*_ for all *kg*, *R*_eff_ reduces to the basic reproduction number. In general this is not the case because *R*_eff_ also takes into account the accumulated immunity at time zero due to prior vaccinations and recovery.

### Pair contact rates

Contacts between individuals are modelled so that $\beta_{gh}/{\hat{N}}_{m}$ denotes the mean contact rate (unnormalized, corresponding to no pandemic) in region *m* between any unordered pair of individuals present in region *m*, such that one individuals is in age group *g* and the other in age group *h*. For *g* ≠ *h* we find that ${\hat{E}}_{gh}^{\left(m\right)}={\hat{N}}_{mg}{\hat{N}}_{mh}$ with the terms on the right given by (6). For *g* = *h*, we note that ${\hat{E}}_{gg}^{\left(m\right)}=EY_{gg}^{\left(m\right)}$ is the expectation of a random integer

$$Y_{gg}^{\left(m\right)}\;=\;\sum_{k=1}^{K}\sum_{1\leq i<j\leq N_{kg}}\;\;\;B_{ki}B_{kj}\;+\sum_{1\leq k<\ell \leq K}\sum_{i=1}^{N_{kg}}\sum_{j=1}^{N_{\ell g}}B_{ki}B_{\ell j},$$

where the random variables *B*_*ki*_ ∈ {0, 1} on the right are mutually independent and such that $EB_{ki}=\theta_{km}$ for all *k*, *i*. Then a direct computation shows that

EYgg(m)=∑k(Nkg2)θkm2+12∑k∑ℓ≠kNkgθkmNℓgθℓm=∑k(Nkg2)θkm2+12(∑k∑ℓNkgθkm)2-∑kNkg2θkm2=12N^mg2-∑kNkgθkm2.

Then the expected number of such pairs equals

$$\begin{array}{c} {\hat{E}}_{gh}^{\left(m\right)}\;=\;\{\begin{array}{cc} {\hat{N}}_{mg}{\hat{N}}_{mh}, & \;g\neq h;\\ \frac{1}{2}{\hat{N}}_{mg}^{2}-\frac{1}{2}\sum_{k}N_{kg}\theta_{km}^{2}, & \;g=h, \end{array} \end{array}$$ (8)

when we assume that each resident of each region *k* is present in region *m* with probability *θ*_*km*_, independently of the other individuals. Then the aggregate rate of contacts between age groups *g* and *h* is given by *β*_*gh*_Γ_*gh*_, where

$$\Gamma_{gh}\;=\;\sum_{m}\frac{{\hat{E}}_{gh}^{\left(m\right)}}{{\hat{N}}_{m}}$$

is a mobility correction factor. The aggregate contact rate between age groups *g* and *h* can alternatively be computed as $\left(1-\frac{1}{2}\delta_{gh}\right)N_{g}C_{gh}$, where *N*_*g*_ is the size of age group *g* and *C*_*gh*_ is the age contact matrix. By solving the balance equation $\left(1-\frac{1}{2}\delta_{gh}\right)N_{g}C_{gh}=\beta_{gh}\Gamma_{gh}$, we find that

$$\begin{array}{c} \beta_{gh}\;=\;\left(1-\frac{1}{2}\delta_{gh}\right)\frac{N_{g}C_{gh}}{\Gamma_{gh}}. \end{array}$$ (9)

For baseline age contact matrix *C*_*gh*_, we use the one in Table C in S1 Appendix, obtained from Finland 2006 POLYMOD matrix, then pairwise degree corrected, then extrapolated and density corrected, then time-corrected to represent an nonnormalised age-based contact structure in Finland in 2021 (assuming no pandemic), see [20].

### Data and initialization

The model is initialized to the epidemic situation in mainland Finland on the day of origin set to 18 April 2021. The age-structured population sizes were retrieved from national statistics [21]. The population sizes per region can be found at Table 3, and further details are in the S1 Appendix. We build an age-dependent contact structure by adjusting a questionnaire-based contact matrix [22] to a setting where the age structure can vary between the geographical regions. Mobility between regions is estimated using aggregate tracking data from a major mobile phone operator.

The disease progression, vaccination, and hospitalization status in the age-region compartments is based mostly on data from Finnish health authorities [23]. With this data we initialize 8 out of 16 compartments for each age-region combination. The compartment related to deaths is set empty, so that the final results only consider new deaths after the initial date. Taking into account all age-region combinations, the model is initialized with 360 values.

### Heuristic vaccination strategies

We construct heuristic vaccination strategies which can depend on three variables for each region *k* and given time *t*: the proportion of population ${\hat{N}}_{k}$, the proportion of new infections ${\hat{I}}_{k}^{D}\left(t\right)$ during the last *D* days, and the proportion of hospitalized individuals ${\hat{H}}_{k}^{D}\left(t\right)$ during the last *D* days in region *k*. Given that there are in total *v*(*t*) vaccine doses to distribute on day *t*, the region *k* will receive

$$\begin{array}{c} v_{k}\left(t\right)=v\left(t\right)(w_{1}{\hat{N}}_{k}+w_{2}{\hat{I}}_{k}^{D}\left(t\right)+w_{3}{\hat{H}}_{k}^{D}\left(t\right)), \end{array}$$ (10)

vaccine doses. Then we distribute the vaccines in each region in an age-descending order, i.e., first vaccinate the entire oldest group, and then the second oldest, etc. The choice of weights *w*_1_, *w*_2_, and *w*_3_ determines the relative allocation of vaccines across regions, with *w*_1_ + *w*_2_ + *w*_3_ = 1. Within regions, the *v*_*k*_(*t*) vaccine doses are distributed in an age-prioritized strategy from older to younger age groups, i.e., first vaccinate the entire oldest group, and then the second oldest, etc. We set *D* = 14 and build 8 different vaccination strategies by setting the *w*_*i*_ values as shown in Table 4. See Section 1 of the S1 Appendix for further details. The feasibility of implementing strategy Pop+Inc+Hosp corresponding to equal weights *w*_1_ = *w*_2_ = *w*_3_ has been discussed by Finnish health authorities [24].

**Table 4:** Adaptive vaccination strategies and their corresponding weights corresponding to (10). Pop, Inc and Hosp refer to strategies where vaccines are distributed demographically, based on the regional incidence level only, and based on the number of hospitalized cases only, respectively.

Strategy	*w*_1_	*w*_2_	*w*_3_
Pop	1	0	0
Inc	0	1	0
Hosp	0	0	1
Pop+Hosp	1/2	0	1/2
Pop+Inc	1/2	1/2	0
Inc+Hosp	0	1/2	1/2
Pop+Inc+Hosp	1/3	1/3	1/3

### Optimized vaccination strategies

In order to obtain an optimized age-specific and time-dependent vaccination strategy, we formulate the problem in terms of optimal control theory with the aim of minimizing the total number of deaths while satisfying the constraints of a fixed daily maximum amount of vaccines available over the course of a single pandemic wave. More specifically, our objective is to determine optimal time-varying-per-capita rate of vaccines *ν* : (*k*, *g*, *t*) ↦ *ν*_*kg*_(*t*) that minimizes the cumulative number of deaths calculated by (1). Thus, the objective functional to be minimized is given by

$$\begin{array}{c} J\left(\nu \right)=\int_{0}^{t_{f}}\sum_{k=1}^{K}\sum_{g=1}^{G}D_{kg}\left(t\right)dt, \end{array}$$ (11)

where the instantaneous expected death rate *D*_*kg*_(*t*) is obtained as a solution of (1)–(4), and *t*_*f*_ is a sufficiently large time instant by which the full population is vaccinated.

The optimal control formulation is: find $\nu^{*}:\left(k,g,t\right)\mapsto \nu_{kg}^{*}\left(t\right)$ such that

$$\begin{array}{c} \begin{array}{cc} & J\left(\nu^{*}\right)=\underset{\nu }{\text{min}}\;J\left(\nu \right)\;\;\;\;\text{subject}\;\text{to}\;\text{(}1\text{)}\;\text{and}\\ & \sum_{k=1}^{K}\sum_{g=1}^{G}\nu_{kg}\left(t\right)S_{kg}\left(t\right)=\nu_{\text{max}}, \end{array} \end{array}$$ (12)

where *ν*_max_ is the maximum rate of available vaccines. To solve this control problem numerically, we use Pontryagin’s Maximum Principle [25, 26]. This principle converts problem (12) into the problem of minimizing the Hamiltonian $H=\sum_{k=1}^{K}\sum_{g=1}^{G}H_{kg}$ given by

$$\begin{array}{c} \begin{array}{cc} H_{kg}\;=\; & D_{kg}\\ & +\Lambda_{S_{kg}^{u}}(-\lambda_{kg}S_{kg}^{u}-v_{kg}S_{kg}^{u})-\Lambda_{S_{kg}^{x}}\lambda_{kg}S_{kg}^{x}\\ & +\Lambda_{S_{kg}^{v}}(v_{kg}S_{kg}^{u}-\lambda_{kg}S_{kg}^{v}-\frac{1}{T_{V}}S_{kg}^{v}))\\ & +\Lambda_{S_{kg}^{p}}(\left(1-e\right)\frac{1}{T_{V}}S_{kg}^{v}-\lambda_{kg}S_{kg}^{p})\\ & +\Lambda_{E_{kg}}(\lambda_{kg}\left(S_{kg}^{u}+S_{kg}^{v}+S_{kg}^{x}\right)-\frac{1}{T_{E}}E_{kg})\\ & +\Lambda_{E_{kg}^{v}}(\left(1-\omega \right)\lambda_{kg}S_{kg}^{p}-\frac{1}{T_{E}}E_{kg}^{v})\\ & +\Lambda_{I_{kg}}(\frac{1}{T_{E}}E_{kg}-\frac{1}{T_{I}}I_{kg})+\Lambda_{I_{kg}^{v}}(\frac{1}{T_{E}}E_{kg}^{v}-\frac{1}{T_{I}}I_{kg}^{v})\\ & +\Lambda_{Q_{kg}^{0}}(\left(1-p_{g}^{h}\right)\frac{1}{T_{I}}I_{kg}+\left(1-\left(1-\pi \right)p_{g}^{h}\right)\frac{1}{T_{I}}I_{kg}^{v}-\frac{1}{T_{Q^{0}}}Q_{kg}^{0})\\ & +\Lambda_{Q_{kg}^{1}}(p_{g}^{h}\frac{1}{T_{I}}\left(I_{kg}+\left(1-\pi \right)I_{kg}^{v}\right)-\frac{1}{T_{Q^{1}}}Q_{kg}^{1})\\ & +\Lambda_{H_{kg}^{w}}(\frac{1}{T_{Q^{1}}}Q_{kg}^{1}\left(t\right)-\frac{1}{T_{H^{w}}}H_{kg}^{w}\left(t\right))\\ & +\Lambda_{H_{kg}^{c}}(p_{g}^{c}\frac{1}{T_{H^{w}}}H_{kg}^{w}-\frac{1}{T_{H^{c}}}H_{kg}^{c})\\ & +\Lambda_{H_{kg}^{r}}(\left(1-\mu_{g}^{c}\right)\frac{1}{T_{H^{c}}}H_{kg}^{c}-\frac{1}{T_{H^{r}}}H_{kg}^{r})\\ & +\Lambda_{R_{kg}}(\left(1-\mu_{g}^{q}\right)\frac{1}{T_{Q^{0}}}Q_{kg}^{0}+\left(1-\mu_{g}^{w}\right)\left(1-p_{g}^{c}\right)\frac{1}{T_{H^{w}}}H_{kg}^{w}+\frac{1}{T_{H^{r}}}H_{kg}^{r})\\ & +\Lambda_{D_{kg}}(\mu_{g}\frac{1}{T_{Q^{0}}}Q_{kg}^{0}+\mu_{g}^{w}\left(1-p_{g}^{c}\right)\frac{1}{T_{H^{w}}}H_{kg}^{w}+\mu_{g}^{c}\frac{1}{T_{H^{c}}}H_{kg}^{c})\\ & +\Lambda_{V_{kg}}(e\frac{1}{T_{V}}S_{kg}^{v}), \end{array} \end{array}$$ (13)

where $\Lambda_{S_{kg}^{u}},\ldots ,\Lambda_{V_{kg}}$ appearing above are time-dependent Lagrange multipliers [27]. Then, we differentiate H with respect to *ν*_*kg*_ to obtain

$$\frac{\partial H}{\partial \nu_{kg}}\left(t\right)\;=\;-(\Lambda_{S_{kg}^{u}}\left(t\right)-\Lambda_{S_{kg}^{v}}\left(t\right))S_{kg}^{u}\left(t\right).$$

Further, we differentiate H with respect to the state variables $S_{kg}^{u}$, $S_{kg}^{v}$, $S_{kg}^{p}$, $S_{kg}^{x}$, *E*_*kg*_, $E_{kg}^{v}$ *I*_*kg*_, $I_{kg}^{v}$, $Q_{kg}^{0}$, $Q_{kg}^{1}$, $H_{kg}^{w}$ $H_{kg}^{c}$, $H_{kg}^{r}$, *R*_*kg*_, *D*_*kg*_, *V*_*kg*_ to derive a so-called adjoint system of equations. By collecting the state variables into a vector $Y=[S_{kg}^{u},\ldots ,V_{kg}]$, and the Lagrange multipliers into a vector $\Lambda_{Y}=\left[\Lambda_{S_{kg}^{u}},\ldots ,\Lambda_{V_{kg}}\right]$, we have

$${\dot{\Lambda }}_{Y}=-\frac{\partial H}{\partial Y},$$

with transversality conditions Λ_*Y*_(*T*_*f*_) = 0. We solve the adjoint system of equations backwards in time because we only have the final conditions. For more details see Section 2 of the S1 Appendix.

## Results

We summarize our results by focusing on the medium-level mobility scenario, i.e., for *τ* = 0.5. The relative performance of different vaccination strategies and their qualitative behavior is robust across different mobility levels (see S1 File).

### Comparison of adaptive heuristic strategies

**Fig 2:**
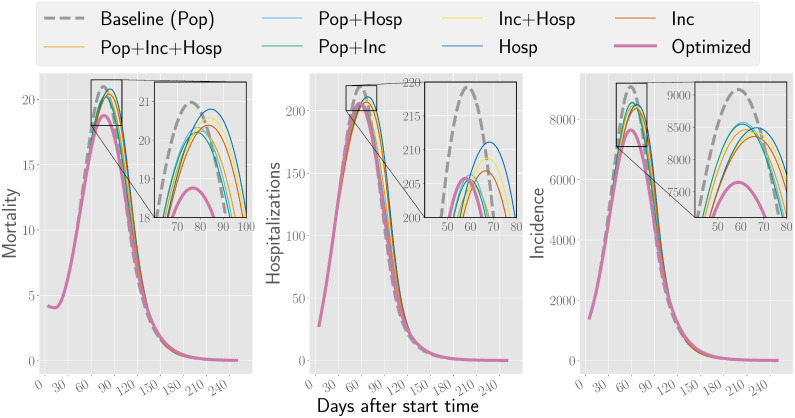
Mortality, hospitalizations, and incidence for the vaccination strategies in Table 2. In this scenario, the effective reproduction number is *R*_eff_ = 1.5 and the mobility value is *τ* = 0.5. For other parameter combinations, see S1 File.

We first compare different vaccination strategies at the level of the whole country (Fig 2) to a baseline strategy, in which vaccine doses are first allocated to regions weighted by population counts and then serially to age groups in descending order within each region. This static baseline strategy Pop differs from all other strategies which we call *adaptive heuristic strategies* in a way that it does not try to adapt to the evolution of the epidemic in any way. The adaptive heuristic strategies allocate more vaccine doses to regions with more infections and/or hospitalizations, but are similarly age-prioritized within regions.

**Fig 3:**
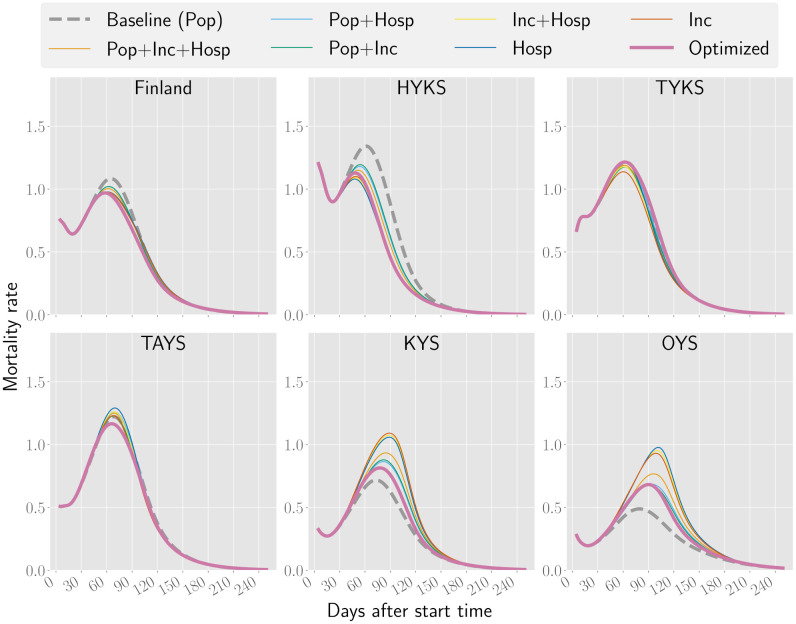
Number of daily deaths per million inhabitants in Finland and the five hospital catchment areas. For this scenario, the basic reproduction number *R*_eff_ = 1.5 and the mobility value *τ* = 0.5. For other values of *R*_eff_ and *τ*, see S1 File.

Fig 2 describes the performance of different strategies over time. All adaptive strategies succeed in lowering incidence compared to the baseline. For mortality and hospitalizations, the heuristic strategies outperform the baseline initially, but tend to lose most of their advantage in the long run. This is because the adaptive heuristics delay the epidemic and its peak as compared to the baseline, and eventually the less-vaccinated regions in the adaptive heuristics will do worse than in the baseline strategy. This can be further seen in Fig 3 which shows the evolution of mortality in each region. In contrast, the optimized strategy succeeds in keeping mortality and hospitalizations below baseline also after the peak. Furthermore, it can be seen in Fig 3 that the optimized strategy leads to a more even distribution of deaths across the regions. This could be potentially beneficial in relieving the pressure on the healthcare system so that not to exceed capacity on hospitals.

Whether or not it pays off to delay the epidemic with adaptive strategies at the cost of allocating less vaccines to less affected regions depends on how fast the disease is progressing. Specifically, the total performance over the full time horizon depends on the transmission rates of the disease (see Table 5): In low-transmission scenarios the adaptive heuristics perform well and delaying the epidemic can be beneficial because there is time to develop additional immunity in the low-incidence regions to hinder future spreading. In high-transmission scenarios the adaptive heuristics put too much emphasis on the initially high-incidence regions and leave the low-incidence regions vulnerable to large future outbreaks.

**Table 5:** Absolute difference in mortality (expected number of deaths) and cumulative incidence (expected number of cases) during a 250-day time horizon resulting from different vaccination strategies with respect to baseline strategy (Pop) for *τ* = 0.5. Highest reductions are indicated in boldface. Results for different values of *τ* are shown in S1 File, including hospitalizations.

	*R*_eff_	Hosp	Inc	Inc+Hosp	Pop+Hosp	Pop+Inc	Pop+Inc+Hosp	Optimized
Mortality	0.75	-0.35	**-0.41**	-0.39	-0.24	-0.27	-0.31	**-0.41**
	1.00	-1.91	-2.41	-2.19	-1.57	-1.79	-1.95	**-3.29**
	1.25	8.55	2.26	5.15	-1.02	-3.71	-0.37	**-18.15**
	1.50	83.77	58.71	70.06	23.19	11.32	33.11	**-57.31**
Incidence	0.75	-405.11	-410.73	-407.56	-214.09	-215.38	-280.55	**-735.16**
	1.00	-1896.11	-2084.31	-1992.86	-1196.29	-1256.36	-1506.76	**-3600.67**
	1.25	257.35	-2649.12	-1269.34	-1906.76	-3052.56	-2138.40	**-20880.99**
	1.50	12984.59	3760.78	8102.05	1065.11	-2905.91	1998.24	**-46701.01**

As expected, none of the strategies can outperform the baseline in every region. The regions that have initially less incidence will suffer on the expense of the high-incidence regions when changing from the baseline strategy to adaptive strategies. However, as stated before, if all individuals in the country are treated equally regardless of their region of residence, the transmission rate will determine which strategy is best for minimizing the total disease-induced mortality in the country.

Among the adaptive vaccination strategies, the number of hospitalized individuals is not in general as good a measure as incidence when determining where to distribute the vaccines. This might be due to the delay in the hospitalization which means that vaccination continues in regions where the effective reproduction number is already low, at the expense of regions where incidence is on the rise but not yet reflected in hospitalizations.

It should be noted that in our model the number of daily new infections is assumed to be accurately reported, which is not a realistic assumption. While it does not make any difference for the strategy if the total numbers are systematically lower due to underreporting, fluctuations in the numbers and systematic biases in the measurements across regions could have an impact.

### Performance of optimized vaccination strategies

We will next discuss the performance of an optimized vaccination strategy found by running the numerical algorithm with the objective of minimizing the total disease-induced mortality over a 250-day time horizon. Our numerical investigations show that the optimization algorithm is robust for different levels of the vaccine efficacy, reduction in susceptibility and protection against severe symptoms, see Section 3 of the S1 Appendix for further details.

Our numerical results indicate that the optimized strategy shares good features of both the static baseline strategy and the adaptive heuristic strategies: There is an initial drop in mortality similar to heuristic strategies, but in the long term the difference to baseline is not as large as for the heuristic strategies. In other words, at later times of the epidemic the optimized strategy demonstrates the highest reduction in mortality. Overall, the optimized strategy shows reduction in mortality by up to 57 individuals for *R*_eff_ = 1.5 (see Table 5). The reason why the differences in mortality are not very large is because the majority of individuals in high-risk groups have already been vaccinated in the beginning of the calculations (18 April 2021). However, cumulative incidence can reach differences of up to tens of thousands, as Table 5 shows.

**Fig 4:**
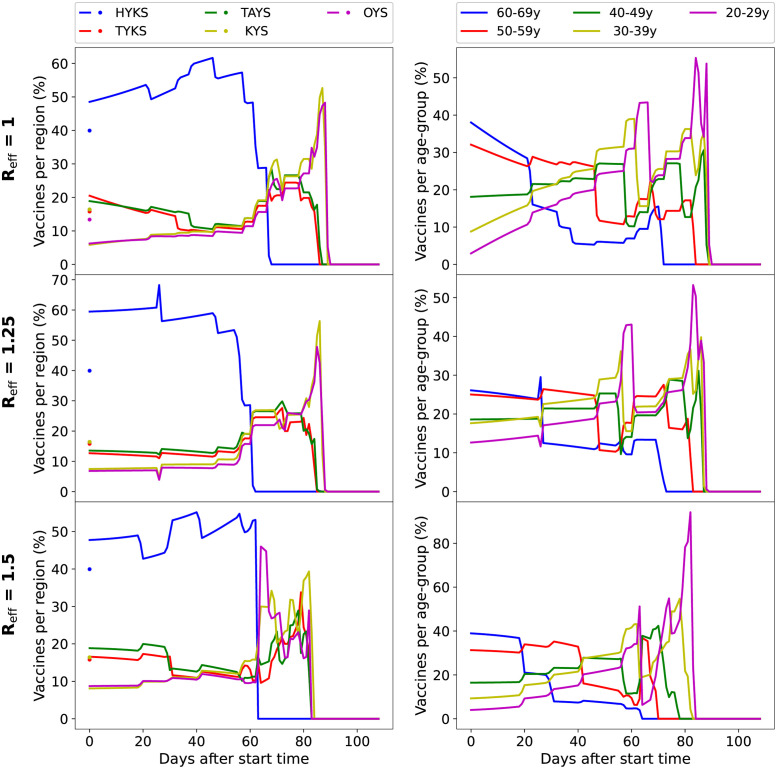
Percentage of vaccine doses allocated by the optimized strategy to regions (left) and age groups (right) in three scenarios (*R*_eff_ = 1, 1.25, 1.5). On the left, dots represent the percentage of vaccines which each region would receive with Pop (baseline).

The percentage of vaccine doses allocated by the optimized strategy to each geographical region and age group is shown in Fig 4 for three transmission scenarios. Similarly to the heuristic strategies, the optimized strategy depends heavily on the disease parameters. The effective reproduction number does not just fine-tune the strategy, but there is a transition from one approach to another: For a low-transmission scenario (*R*_eff_ = 0.75) in which the epidemic is in clear decline, the optimized strategy does not preferentially target older age groups but tries to reduce the number of infections, and the optimized strategy is the one that follows the number of infected. In scenarios with a high overall transmission rate, the optimized strategy favours older age groups having higher risk of severe illness and death.

Both the low-transmission and high-transmission scenarios lead to an optimized strategy that favours the initially high-incidence region, and this effect is stronger for low-transmission scenarios. Specifically, the optimized strategy initially targets the capital region (HYKS) with approximately 20 (resp. 8) percentage points higher share of available vaccine doses than the baseline strategy for *R*_eff_ = 1.25 (resp. 1.5). Interestingly, the optimization finds that the age prioritization is smaller and geography prioritization more aggressive in the scenario with *R*_eff_ = 1.25 than in scenarios with *R*_eff_ = 1.0 and *R*_eff_ = 1.5.

## Discussion and conclusions

In this work we have constructed an epidemic modelling framework which allows to evaluate various adaptive strategies for allocating vaccines based on static demographic data and dynamic evolution of the epidemic situation across different geographical regions. We investigated various heuristic strategies for allocating more vaccines to regions with higher incidence and hospital load, together with optimized strategies which may flexibly allocate vaccines to different age groups and regions in parallel. Our numerical results, conducted for scenarios adjusted to the recent COVID-19 epidemic situation in Finland, show that optimized vaccination strategies can reduce the death toll and significantly mitigate the disease burden of the epidemic. The relative advantage of different adaptive strategies over the static baseline is influenced by the overall epidemic situation. Also, whatever strategy is chosen, a trade-off between different regions is inevitable due to limited supply of vaccine doses and daily vaccination capacity. Nevertheless, the results provide valuable insights for designing efficient vaccination strategies: In general, using hospital loads as basis in allocating vaccine doses tends to lead to worse performance compared to the static baseline. The optimized strategy appears to achieve a good balance between short-term benefits of adaptive strategies and the long-term robustness gained by the uniform vaccine allocation. Further, even though we optimize mortality, there is a delicate balance between favoring individuals with higher direct risk of death as opposite to individuals at risk of getting infected and causing large outbreaks.

As with all modelling, there are several factors and phenomena that are not included, and the results can change if these factors turn out to be important. Typically this would imply that the actual numbers in a modelling study might be subject to change, but the overall phenomena that are observed here are relatively robust. Such numbers would be the exact number of infected, hospitalized, and deceased individuals, and the phenomena the relative order of the different strategies. The only real way of knowing which factors are important is to include them in a model, but in practice the choice of relevant factors is informed by the reliability of the model. This is why we have chosen to start with a model benchmarked in another study related to Sweden [12], and modify it by making it more accurate by including geographical information.

There are several factors which we believe that are missing in our model and are important for both the accuracy of the results and important to consider when optimizing vaccination strategies. First is the need for more than a single vaccine dose needed by many of the currently used vaccines, which is not modelled here. Including this in the model would allow one to optimize the vaccination strategy further by finding an optimal strategy to give the second dose with relation to vaccinating different age groups and geographical locations, or to identify the optimal time delay between the two doses [28]. This could have an impact on the benefits of regional targeting strategies, because the regional differences might even out during the time it takes to build immunity with multiple vaccines. Second, one should allow the infectious contact rates to change across geographical regions and time. As the public is informed of the current pandemic situation their behavior, and therefore the transmission rate, is bound to change. This induces a feedback loop which makes a large difference especially for long-term predictions, but also makes modeling more difficult as one needs to model the public response to various pandemic situations [29, 30]. In addition, the governments will take actions given that the situation is sufficiently critical [31], and these decisions might depend on several hard-to-model factors related to politics.

Studying the effects of cross-region mobility were not at the main focus of this study, but the sensitivity analysis that we performed for the overall mobility factor has interesting implications. It turned out that cross-region mobility can be an important factor even in this relatively advanced state of the epidemic where all regions have some incidence, but there is still a geographical imbalance in the relative incidences. These results are especially striking considering that the mobility factor *τ* only controls for cross-region mobility but not the overall contact rates of the individuals. That is, decreasing *τ* decreases the cross-region contacts but increases the inside-region contacts, and the total rate of contacts in the country remains the same but the large-scale geographical mixing patterns changed. This is in contrast to conventional models which assume full mixing across the country. Further, these findings could have implications on interventions that limit long-range mobility. Further research in this direction would be needed for concluding about these type of interventions.

Our analysis reveals that designing efficient vaccination strategies at a level of a country is highly nontrivial. As seen from our results in Fig 4, the details of optimized strategies can be complicated and their faithful implementation difficult, and could lead in a slower overall vaccine delivery. However, it should be possible to simplify the strategies and try to follow the main principles of parallel vaccination and geographic distribution of vaccines with as much detail as practically possible. It is important to note that carefully analyzed and executed strategies can potentially save lives even if the strategy is changed after most of the risk groups are already vaccinated. Much larger effects could potentially be obtained if the planning were done before vaccinations started, but in this case the problem is that the various parameters related to vaccination efficiency might not be known. In any case, the relative performance of different strategies can depend on the effective reproduction number, which means that the vaccination strategy should be chosen in conjunction with non-pharmaceutical intervention strategies of the country.

## Supporting information

S1 Appendix Detailed description of the heuristic strategies, the optimization algorithm, data, and initial conditions. **Table B**. Finnish age contact matrix with 9 age groups and 10y age resolution. **Table C**. Finnish regional morning (between 6:00–11:59) mobility, averaged over March–May 2019.
(PDF)

S1 File Additional numerical results. (PDF)
