# Supplementary material for: Adaptive and optimized COVID-19 vaccination strategies across geographical regions and age groups
Source: PLoS Comput Biol. 2022 Apr 7;18(4):e1009974. doi: 10.1371/journal.pcbi.1009974 (PMC9017881; doi:10.1371/journal.pcbi.1009974)
Supplement: S1 Appendix — Table B. Finnish age contact matrix with 9 age groups and 10y age resolution. Table C. Finnish regional morning (between 6:00–11:59) mobility, averaged over March–May 2019. (PDF) [file pcbi.1009974.s001.pdf]

Supplementary information

1 Heuristic vaccination strategies

We first analyse the different heuristic vaccination strategies to asses their impact on the development of the epidemic. Specifically, we construct different scenarios that determine the number of vaccines $v_k(t)$ that each region k will receive on day t depending on the number of infections and/or hospitalizations. Then, the vaccines $v_k(t)$ are distributed within the region in an age-prioritized strategy from old to younger age groups. We can obtain $v_k(t)$ in the following way

$$v_k(t) = v(t)\Big(w_1 \frac{N_k}{\sum_k N_k} + w_2 \frac{I_k^D(t)}{\sum_k I_k^D(t)} + w_3 \frac{H_k^D(t)}{\sum_k H_k^D(t)}\Big),$$

where $v(t)$ is the overall national number of available vaccine doses on day t, w_1, w_2, and w_3 are tunable weight parameters of the strategy ($\sum_i w_i = 1$), $I_k^D(t)$ is the number of new infections, and $H_k^D(t)$ is the total hospital occupation in region k over the last D days. In our work we set $D = 14$ to capture the changes over two weeks starting from the initial date. The total number of new infections in region k in the last D days is computed by

$$I_k^D(t) = \int_{t-D}^{t} \sum_{g=1}^{G} \frac{1}{T_E} E_{kg}(t)dt,$$

and similarly the number of hospitalized individuals is computed by

$$H_k^D(t) = \int_{t-D}^{t} \sum_{g=1}^{G} \Big(H_{kg}^w(t) + H_{kg}^c(t) + H_{kg}^r(t)\Big)dt.$$

Note that, since we want $H_k^D(t)$ to reflect the total hospital occupation at time t for region k, an individual may be counted more than once. One at day t, another at $t-1$ and so on. As long as the individuals remain in the hospital they will be counted.

Different vaccination strategies can be obtained by changing the weights w_i, e.g. setting $w_1 = 1$ and $w_2 = w_3 = 0$ corresponds to the baseline strategy `Pop` where vaccines are equally distributed according to the population density.

2 Numerical discretization of optimal control problems

In this section we describe the numerical approach to solving a general optimal control problem via Pontryagin's Maximum Principle [1, 2].

Let $\mathbf{x}(t) = [x_1(t),\, x_2(t),\, \ldots,\, x_n(t)]^\mathsf{T} \in \mathbb{R}^n$ be a state vector and $\mathbf{u}(t) = [u_1(t),\, u_2(t),\, \ldots,\, u_r(t)]^\mathsf{T} \in \mathbb{R}^r$ be a control vector. Consider the following optimal control problem: find $\mathbf{u}(t)$ to minimize

$$J(\mathbf{u}) = \int_0^{t_f} g(\mathbf{x}(t), \mathbf{u}(t), t)dt \tag{2.1}$$

subject to the state equations

$$\dot{\mathbf{x}}(t) = f(\mathbf{x}(t), \mathbf{u}(t), t), \quad \mathbf{x}(0) = \mathbf{x}_0 \tag{2.2}$$

and the constraint

$$\mathbf{a} \leq \mathbf{u}(t) \leq \mathbf{b}.$$

In order to state the Maximum Principle we define a Hamiltonian as

$$\mathcal{H}(\mathbf{x}(t), \mathbf{u}(t), t) = g(\mathbf{x}(t), \mathbf{u}(t), t) + \mathbf{q}^\mathsf{T}(t) f(\mathbf{x}(t), \mathbf{u}(t), t), \tag{2.3}$$

where $\mathbf{q}(t)$ are the time-dependent Lagrange multipliers [1]. The goal now is to find an optimal trajectory $\mathbf{x}(t)$, an optimal control $\mathbf{u}(t)$ and an optimal set of Lagrange multipliers $\mathbf{q}(t)$ so that to minimize the objective function in (2.1).

From the Hamiltonian (2.3) and Pontryagin's Maximum Principle, we obtain the following theorem [1].

Theorem 1. *If $\mathbf{x}^*(t), \mathbf{u}^*(t), t \in [0, t_f]$ is an optimal state-control trajectory starting at $\mathbf{x}(0)$, then there exist Lagrange multipliers $\mathbf{q}^*(t)$ such that*

$$\begin{aligned}
\dot{\mathbf{x}}(t) &= \frac{\partial \mathcal{H}}{\partial \mathbf{q}} = f(\mathbf{x}(t), \mathbf{u}(t), t), \quad \mathbf{x}(0) = \mathbf{x}_0, \\
-\dot{\mathbf{q}}(t) &= \frac{\partial \mathcal{H}}{\partial \mathbf{x}} = g_x(\mathbf{q}(t), \mathbf{u}(t), t) + \mathbf{q}^\mathsf{T}(t) f_x(\mathbf{q}(t), \mathbf{u}(t), t), \quad \mathbf{q}(t_f) = 0, \\
\mathcal{H}(x^*(t), u^*(t), q^*(t), t) &= \arg\min_{u(t)} \mathcal{H}(x^*(t), u(t), q^*(t), t).
\end{aligned} \tag{2.4}$$

For a given initial value $x_0 \in \mathbb{R}^n$, the numerical approach now consists of finding functions $\mathbf{x} : [0, t_f] \mapsto \mathbb{R}^n$, $\mathbf{u} : [0, t_f] \mapsto \mathbb{R}^n$ and $\mathbf{q} : [0, t_f] \mapsto \mathbb{R}^n$ satisfying the optimality system (2.4). The numerical algorithm consists of the following steps:

Step 1: Subdivide the interval $[0, t_f]$ into N equal sub-intervals and assume a piecewise-constant control $\mathbf{u}^{(0)}(t) = \mathbf{u}^{(0)}(t_k)$, $t \in [t_k, t_{k+1}]$, $k = 0, 1, \ldots, N-1$

Step 2: Integrate the state equations forward in time for the assumed control $\mathbf{u}^{(i)}$ and store the trajectory $\mathbf{x}^{(i)}$

Step 3: Compute $\mathbf{q}^{(i)}$ by solving the second equation in (2.4) backwards in time

Step 4: Compute a new control $\mathbf{u}^{i+1}$ by solving a finite-dimensional nonlinear optimization problem using a sequential least squares programming algorithm

Step 5: Compute $\mathbf{x}^{(i+1)}$ and $\mathbf{q}^{(i+1)}$ for the new control variable as in Steps 2 and 3

Step 6: Compute the values $J^{(i)}(\mathbf{u}^{(i)}, \mathbf{x}^{(i)})$ and $J^{(i+1)}(\mathbf{u}^{(i+1)}, \mathbf{x}^{(i+1)})$

Step 7: If

$$|J^{(i+1)} - J^{(i)}| \leq \epsilon \tag{2.5}$$

stop the iterative procedure. Here ϵ is a small positive constant used as a tolerance. If (2.5) is not satisfied, replace $\mathbf{u}^{(i)}$ with $\mathbf{u}^{(i+1)}$, $\mathbf{x}^{(i)}$ with $\mathbf{x}^{(i+1)}$, $\mathbf{q}^{(i)}$ with $\mathbf{q}^{(i+1)}$ and return to Step 4.

3 Different type of vaccines

In this section we investigate the sensitivity of the optimization algorithm to different levels of the vaccine efficacy e, reduction in susceptibility ω and protection against developing severe illness π. We set $e = 0.5, 0.7, 0.9$, $\omega = 0, 0.2, 0.6$ and $\pi = 0, 0.2, 0.6$ and test the robustness of the optimized strategies with respect to different combinations of these parameters. Let

$$P = \{0.5, 0.7, 0.9\} \times \{0.0, 0.2.0.6\} \times \{0.0, 0.2.0.6\} \tag{3.1}$$

be a Cartesian product representing the set of all combinations for the different values of the parameters e, ω, π. Let S_k be the optimized strategy obtained for $P_k \in P$, $k = 1, 2, \ldots, 27$. We investigate the difference between the optimized vaccination strategies, i.e.,

$$\sigma_{kl} = D(P_k, S_k) - D(P_k, S_l), \quad k, l = 1, 2, \ldots, 27,$$

where D is the total number of deaths. Further, we choose the value of the mobility parameter $\tau = 0.5$.Taking the max norm of the $\mathbf{\Sigma} = (\sigma_{kl}) \in \mathbb{R}^{27\times 27}$ matrix, we get

$$\|\Sigma\|_{\max} = \max_{kl}(|\sigma_{kl}|) = 0.82.$$

Hence the difference in the total number of deaths is less than 1 individual and the performance of the optimized strategies is the same for combinations for the different values of the parameters. Further, Figs M and N in S2 file verify that the optimization algorithm is robust to different values of the parameters e, ω, π since the optimized strategies are similar. The algorithm is expected to be robust against changes in the model parameters concerning the efficacy of the vaccine or protection of the vaccine against severe illness since the gradient of the optimization algorithm does not explicitly depend on these parameters.

4 Data and parameters

4.1 Demographic data for Finland

4.2 Initial conditions

We obtain the initial conditions from data trying to mimic the pandemic situation in Finland as of 18 April 2021. More specifically, we calculate the initial conditions for the compartments in Table D

	0–9	10–19	20–29	30–39	40–49	50–59	60–69	70–79	80+	Total
HYKS	221613	238313	272674	316173	285988	289128	256006	212089	106198	2198182
TYKS	82812	93001	103572	106093	101979	111874	113383	99917	56373	869004
TAYS	88071	100864	105275	112809	106951	115157	117896	100045	55613	902681
KYS	71910	84213	92466	91390	85302	103387	119723	95591	53252	797234
OYS	80308	91471	84511	88448	82348	91225	100322	75669	42261	736563
Total	544714	607862	658498	714913	662568	710771	707330	583311	313697	5503664

Table A: Population size by region and age in mainland Finland on 31 Dec 2020. Obtained from [3].

	0–9	10–19	20–29	30–39	40–49	50–59	60–69	70–79	80+
0–9	4.61	1.24	0.81	1.71	1.08	0.63	0.58	0.15	0.08
10–19	1.10	7.83	0.97	1.02	1.83	0.71	0.35	0.13	0.07
20–29	0.71	0.95	3.87	1.84	1.51	1.41	0.67	0.19	0.10
30–39	1.51	1.01	1.86	3.25	2.24	1.97	1.18	0.21	0.12
40–49	0.82	1.57	1.33	1.94	3.18	2.24	1.02	0.29	0.16
50–59	0.45	0.57	1.16	1.60	2.09	2.91	1.71	0.26	0.14
60–69	0.62	0.42	0.83	1.43	1.42	2.56	2.19	0.72	0.40
70–79	0.22	0.21	0.32	0.36	0.58	0.55	1.01	1.09	0.60
80+	0.22	0.21	0.32	0.36	0.58	0.55	1.01	1.09	0.60

Table B: Finnish age contact matrix with 9 age groups and 10y age resolution. The entry on row g and column h indicates the estimated daily number of contacts made by a typical individual in age group g to individuals in age group h [4].

as follows

$$
\begin{aligned}
S^u_{kg} &= N_{kg} - S^v_{kg} - S^x_{kg} - E_{kg} - I_{kg} - Q^0_{kg} - Q^1_{kg} - H^w_{kg} - H^c_{kg} - H^r_{kg} - R_{kg} - D_{kg} - V_{kg} \\
S^v_{kg} &= 0 \\
S^x_{kg} &= (1-e)v_{kg} \\
E_{kg} &= \frac{T_E}{T_I + T_E} i^r_{kg} \\
I_{kg} &= \frac{T_I}{T_I + T_E} i^r_{kg} \\
Q^0_{kg} &= 0 \\
Q^1_{kg} &= 0 \\
H^w_{kg} &= h_k \mathcal{H}_g \\
H^c_{kg} &= c_k \mathcal{C}_g \\
H^r_{kg} &= 0 \\
R_{kg} &= r^r_{kg} \\
D_{kg} &= 0 \\
V_{kg} &= e\ v_{kg},
\end{aligned}
$$

where v_{kg} is the cumulative number of people who have received the first dose of any vaccine until 18 April 2021, i^r_{kg} stands for the estimated number of real infectious, r^r_{kg} represents the number of real recovered people as of 18 April, h_k is the reported individuals in hospital ward, c_k is the reported individuals in critical care units, and $\mathcal{H}_g$ and $\mathcal{C}_g$ are the proportions of people at ward

	HYKS	TYKS	TAYS	KYS	OYS
HYKS	1 389 016	7 688	16 710	7 789	1 774
TYKS	11 316	518 173	14 139	562	2 870
TAYS	22 928	12 404	511 506	4 360	1 675
KYS	8 990	365	4 557	459 867	3 286
OYS	1 798	2 417	1 592	3 360	407 636

Table C: Finnish regional morning (between 6:00–11:59) mobility, averaged over March–May 2019. Rows represent origins and columns represent destinations [5].

Table D: **Epidemiological compartments.** There are KG copies of each compartment, denoted $S^u_{kg}, S^v_{kg}, \ldots, V_{kg}$ for regions $k = 1, \ldots, K$ and age groups $g = 1, \ldots, G$.

**Symbol**	**Description**
S^u	Susceptible, unvaccinated
S^v	Susceptible, invited for vaccination
S^x	Susceptible, vaccinated with no immunity or declined vaccination
E	Infected but not yet infectious
I	Infected and infectious
Q^0	Quarantined at home, mild disease
Q^1	Quarantined at home, severe disease
H^w	hospitalized, in general ward
H^c	hospitalized, in critical care
H^r	hospitalized, in recovery ward
D	Deceased
R	Recovered with full immunity
V	Vaccinated with full immunity

and critical care, respectively. The estimation of real infectious individuals at any day t is derived directly from data as follows:

$$i^r_{kg}(t) = i^d_{kg}(t) + i^u_{kg}(t),$$

where the number of undetected infectious people $i^u_{kg}(t)$ come from upscaling the number of detected individuals $i^d_{kg}(t)$ by a factor that depends on the index of age group g, i.e.,

$$i^u_{kg}(t) = (1 + 9g^{-2.46}) i^d_{kg}(t).$$

The number of detected infectious people is calculated by summing the reported cases over the last $T_I + T_E$ days,

$$i^d_{kg}(t) = \sum_{\omega=\omega_0}^{t} i^d_k(\omega) \mathcal{I}^w_g(\omega),$$

where $\omega_0 = t - T_I - T_E$, $i^d_k(t)$ is the number of cases in region k reported by THL (Finnish Institute of Health and Welfare) at day t, and $\mathcal{I}^w_g(t)$ is the proportion of infected people in age group g. We do not have daily counts as THL does not provide these on infected people per age group. We have

chosen 18 April 2021 as the start day since it is a Sunday, and the weekly proportion $\mathcal{I}_g^w(t)$ is the same for all the sums ($T_I + T_E = 7$ days, 1 week), which gives

$$i_{kg}^d(t) = \mathcal{I}_g^w \sum_{\omega=\omega_0}^{t} i_k^d(\omega).$$

The numerical value for $\mathcal{I}_g^w$ can be found in Table E and for the result of the summation $\sum_\omega i_k^d(\omega)$ see Table F. The estimation of real recovered people at day t is similar,

$$\begin{aligned} r_{kg}^r(t) &= r_{kg}^d(t) + r_{kg}^u(t) \\ r_{kg}^u(t) &= (1 + 9g^{-2.46}) r_{kg}^d(t) \\ r_{kg}^d(t) &= \sum_{\omega=0}^{t-(T_I+T_E)} i_k^d(\omega) \mathcal{I}_g^w(\omega) \end{aligned}$$

in which $\omega = 0$ marks the beginning of the coronavirus epidemic in Finland. The estimated values at 18 April 2021 of the real infected people i_{kg}^r and real recovered people r_{kg}^r can be found in Tables I and J, respectively.

**Parameter**	**Description**	0–9	10–19	20–29	30–39	40–49	50–59	60–69	70–79	80+
$\mathcal{H}_g$*	Proportion in ward	0.0058	0.0107	0.0467	0.0605	0.0911	0.1450	0.1547	0.2008	0.2847
$\mathcal{C}_g$*	Proportion in critical care	0.0038	0.0069	0.0301	0.0390	0.0978	0.2231	0.2891	0.2448	0.0655
$\mathcal{I}^w$**	Infections	240	310	354	355	294	200	101	44	31
$\mathcal{I}_g^w$	Normalized $\mathcal{I}^w$	0.1244	0.1607	0.1835	0.1840	0.1524	0.1037	0.0524	0.0228	0.0161

Table E: Parameters for age compartments.
* From [6].
** Reported number of infected people in Finland by age group during 12–18 April 2021 [7].

**Parameter**	**Description**	HYKS	TYKS	TAYS	KYS	OYS	Total
h_k*	Ward	88	11	17	5	11	132
c_k*	Critical care	21	6	2	5	0	34
$\sum_\omega i_k^d(\omega)$**	Infectious	1179	347	225	80	76	1907

Table F: Parameters estimated from data
* Numbers reported by [8] on 19 April 2021.
** Sum of reported number of infected people by region from 12–18 April 2021 [7].

v_{kg}	HYKS	TYKS	TAYS	KYS	OYS	Total	Total/N_g (%))
0–9	0	0	0	0	0	0	0
10–19	1802	895	647	467	397	4208	0.69
20–29	14326	6391	4806	4111	3570	33204	5.04
30–39	22284	8958	7639	6314	5640	50835	7.11
40–49	32713	12418	11718	8261	7842	72952	11.01
50–59	53123	20671	20143	15545	14676	124158	17.47
60–69	111319	46461	47329	40640	33953	279702	39.54
70–79	184419	87350	85498	79872	63631	500770	85.85
80+	94809	50321	49239	47561	37125	279055	88.96
Total	514795	233465	227019	202771	166834	1344884	24.44
Total/N_k (%)	23.42	26.87	25.15	25.43	22.65	24.44	

Table G: Number of vaccinated people in Finland by region with 9 age groups and 10y age resolution as of 18 April 2021. The entry on row g and column k indicates the number of individuals who have received the first dose in age group g and region k. Data from [7].

S^u_{kg}	HYKS	TYKS	TAYS	KYS	OYS	Total	Total/N_g (%)
0–9	171581.02	70816.88	81903.12	67257.71	76718.73	468277.46	85.97
10–19	209955.74	85708.42	96837.14	80990.48	88887.55	562379.33	92.52
20–29	228534.80	89876.46	96665.64	85189.05	78392.50	578658.45	87.88
30–39	270855.73	91783.82	102310.14	82761.97	80860.11	628571.77	87.92
40–49	235429.06	85427.65	93018.80	75258.19	73005.88	562139.57	84.84
50–59	221934.99	88013.76	93279.21	86411.78	75323.60	564963.33	79.49
60–69	137785.90	65351.37	69714.24	78372.62	65760.56	416984.69	58.95
70–79	24360.73	11823.42	14133.96	15379.26	11743.93	77441.30	13.28
80+	8689.93	5505.76	6064.27	5424.06	4885.78	30569.80	9.75
Total	1509127.92	594307.53	653926.52	577045.10	555578.63	3889985.71	70.68
Total/N_k (%)	68.65	68.39	72.44	72.38	75.43	70.68	

Table H: Number of susceptible people in Finland by region with 9 age groups and 10y age resolution as of 18 April 2021. The entry on row g and column k indicates the number of individuals who are susceptible in age group g and region k.

i^r_{kg}	HYKS	TYKS	TAYS	KYS	OYS	Total	Total/N_g (%)
0–9	1613.56	473.53	307.93	110.86	102.64	2608.52	0.48
10–19	688.86	202.16	131.46	47.33	43.82	1113.63	0.18
20–29	563.26	165.30	107.49	38.70	35.83	910.58	0.14
30–39	498.45	146.28	95.12	34.24	31.71	805.81	0.11
40–49	390.24	114.52	74.47	26.81	24.82	630.87	0.10
50–59	257.88	75.68	49.21	17.72	16.40	416.90	0.06
60–69	128.09	37.59	24.45	8.80	8.15	207.08	0.03
70–79	55.24	16.21	10.54	3.80	3.51	89.30	0.02
80+	38.66	11.35	7.38	2.66	2.46	62.50	0.02
Total	4234.25	1242.62	808.06	290.90	269.35	6845.19	0.12
Total/N_k (%)	0.19	0.14	0.09	0.04	0.04	0.12	

Table I: Estimated number of real infectious people in Finland by region with 9 age groups and 10y age resolution as of 18 April 2021. The entry on row g and column k indicates the number of individuals who are infectious in age group g and region k.

r_{kg}^{r}	HYKS	TYKS	TAYS	KYS	OYS	Total	Total/N_g (%)
0–9	48404.26	11519.76	5855.94	4539.03	3486.14	73805.15	13.55
10–19	25865.12	6195.55	3247.81	2708.32	2142.57	40159.38	6.61
20–29	29244.78	7138.85	3694.80	3127.12	2512.22	45717.78	6.94
30–39	22527.03	5203.81	2763.23	2279.31	1915.45	34688.83	4.85
40–49	17445.50	4017.41	2137.80	1755.16	1474.33	26830.20	4.05
50–59	13794.60	3110.75	1682.52	1410.74	1207.43	21206.04	2.98
60–69	6751.83	1529.35	824.89	699.27	598.56	10403.91	1.47
70–79	3231.19	723.73	398.56	333.74	288.36	4975.57	0.85
80+	2633.95	531.39	297.37	262.54	244.63	3969.89	1.27
Total	169898.27	39970.61	20902.92	17115.24	13869.71	261756.74	4.76
Total/N_k (%)	7.73	4.60	2.32	2.15	1.88	4.76	

Table J: Estimated number of real recovered people in Finland by region with 9 age groups and 10y age resolution as of 18 April 2021. The entry on row g and column k indicates the number of individuals who are recovered in age group g and region k.

References

1. Kirk DE. Optimal control theory: An introduction. Courier Corporation; 2004.

2. Macki J, Strauss A. Introduction to Optimal Control Theory. Springer; 2012.

3. Official Statistics of Finland. Population structure database; 2021. Available from: https://pxnet2.stat.fi/PXWeb/pxweb/en/StatFin/StatFin__vrm__vaerak/statfin_vaerak_pxt_11re.px/.

4. Mossong J, Hens N, Jit M, Beutels P, Auranen K, Mikolajczyk R, et al. Social Contacts and Mixing Patterns Relevant to the Spread of Infectious Diseases. PLOS Medicine. 2008;5(3). doi:10.1371/journal.pmed.0050074.

5. Telia Crowd Insights. Website: https://www.telia.fi/yrityksille/palvelut/teknologiat/crowd-insights.

6. Sjödin H, Rocklöv J, Britton T. Evaluating and optimizing COVID-19 vaccination policies: A case study of Sweden. medRxiv. 2021;doi:10.1101/2021.04.07.21255026.

7. Finnish Institute for Health and Welfare (THL). COVID-19 API; 2021. Available from: https://sampo.thl.fi/pivot/prod/en/epirapo/covid19case/fact_epirapo_covid19case. Instructions: https://thl.fi/en/web/thlfi-en/statistics/statistical-databases/open-data/confirmed-corona-cases-in-finland-covid-19-.

8. Sanomat H. Helsingin Sanomat, Hospitalized data API; 2021. Available from: https://w3qa5ydb4l.execute-api.eu-west-1.amazonaws.com/prod/finnishCoronaHospitalData. Repository: https://github.com/HS-Datadesk/koronavirus-avoindata.
