# Supplementary material for: Adaptive and optimized COVID-19 vaccination strategies across geographical regions and age groups
Source: PLoS Comput Biol. 2022 Apr 7;18(4):e1009974. doi: 10.1371/journal.pcbi.1009974 (PMC9017881; doi:10.1371/journal.pcbi.1009974)
Supplement: S1 File — (PDF) [file pcbi.1009974.s002.pdf]

Time plots for different values of mobility levels

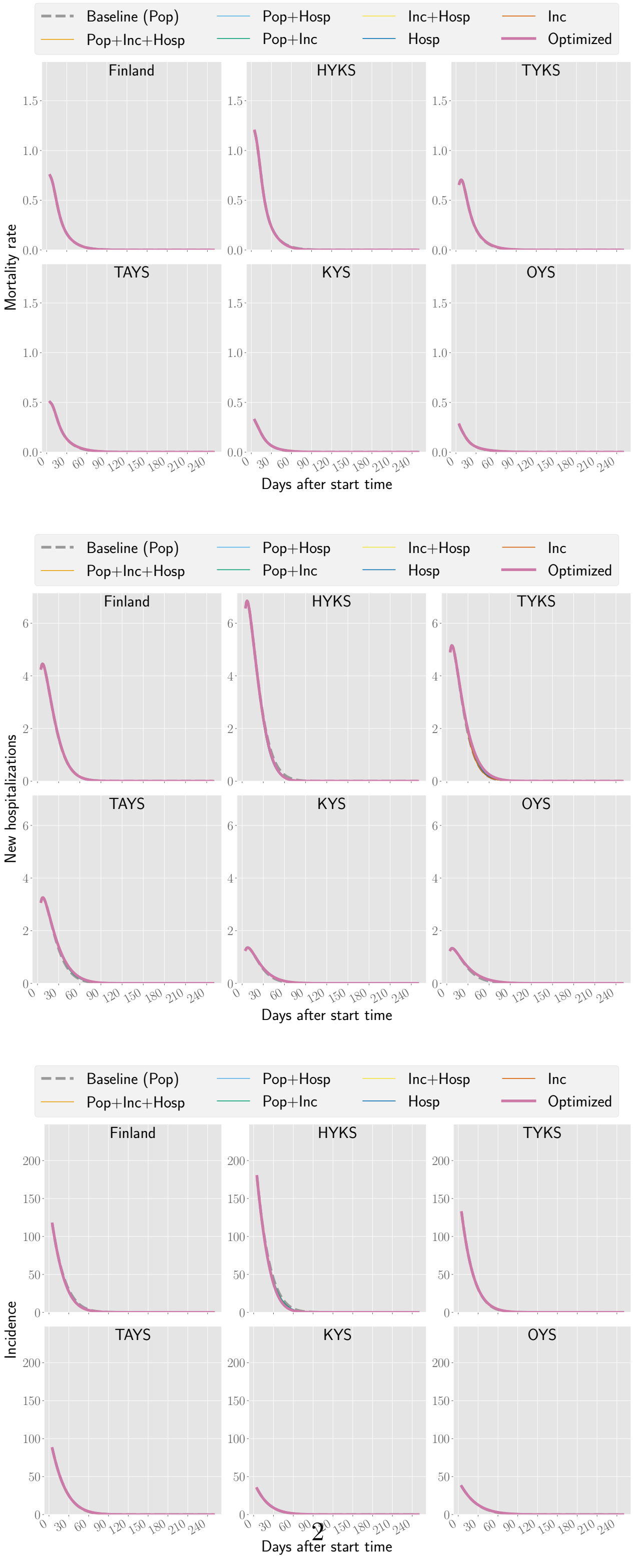

Fig. A: Different metrics per million inhabitants in Finland and the five hospital catchment areas For these scenarios, the basic reproduction number $R_{\text{eff}} = 0.75$ and the mobility value $\tau = 0.0$.

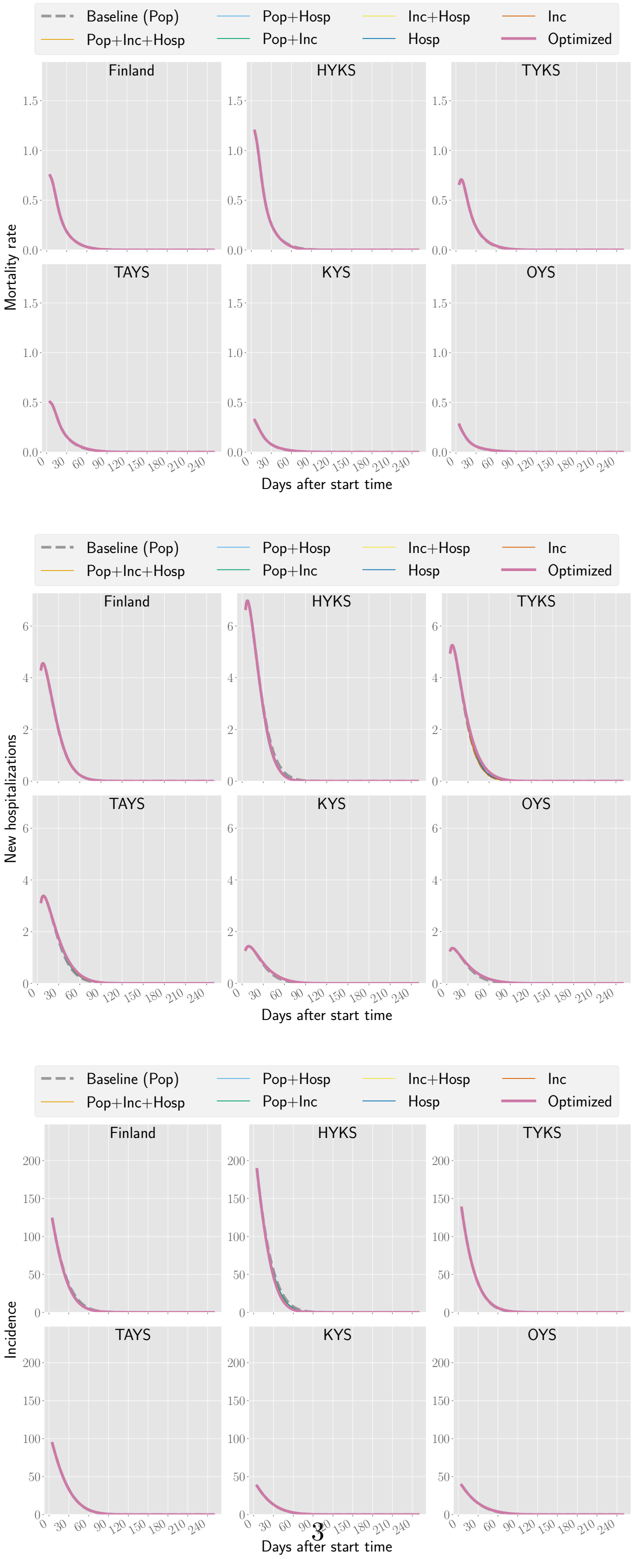

Fig. B: Different metrics per million inhabitants in Finland and the five hospital catchment areas included here for all the vaccination strategies. For these scenarios, the basic reproduction number $R_{\text{eff}} = 0.75$ and the mobility value $\tau = 0.5$.

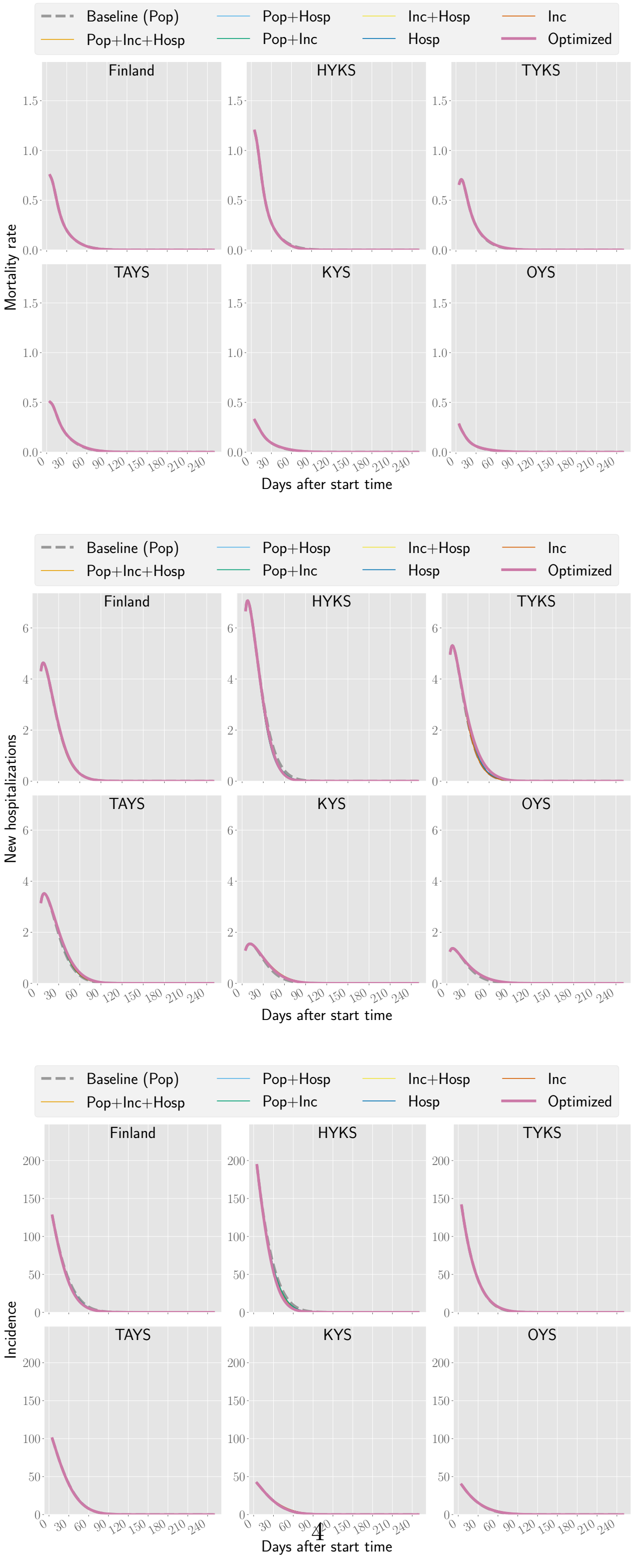

Fig. C: Different metrics per million inhabitants in Finland and the five hospital catchment areas included here for all the vaccination strategies. For these scenarios, the basic reproduction number $R_{\text{eff}} = 0.75$ and the mobility value $\tau = 1.0$.

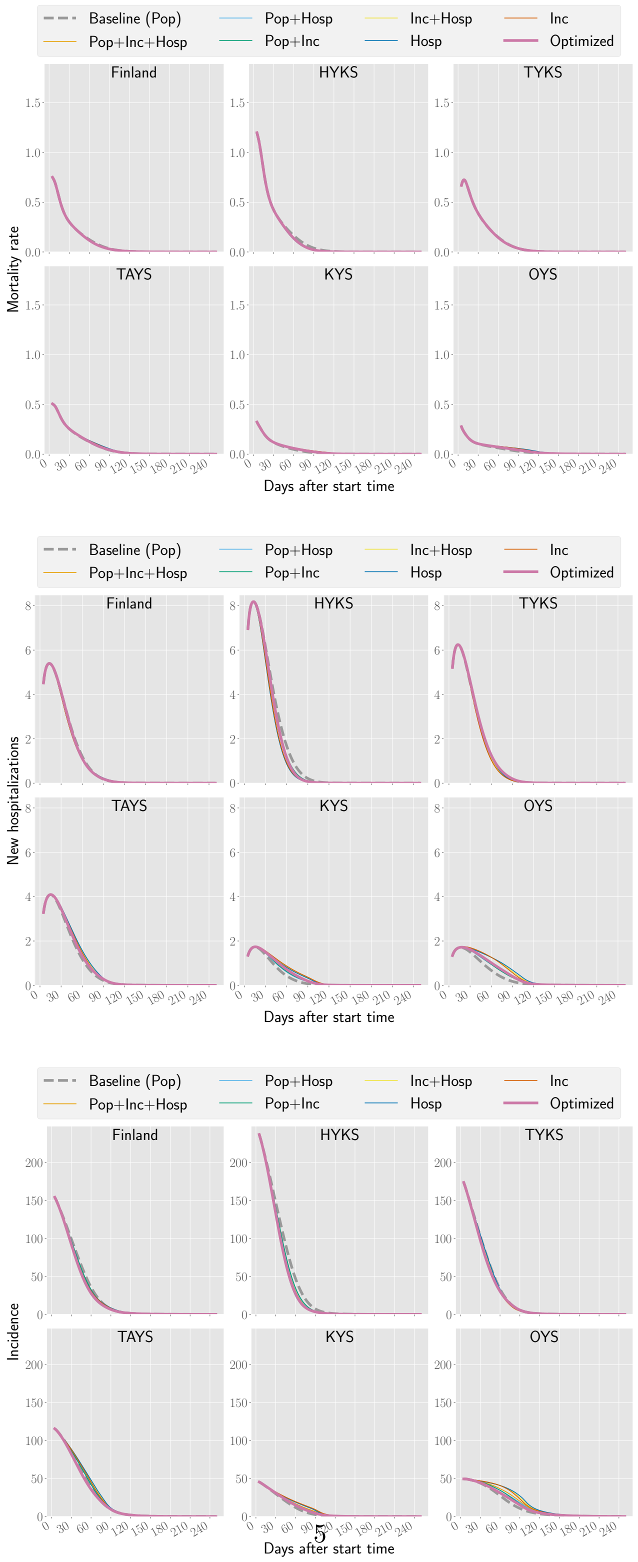

Fig. D: Different metrics per million inhabitants in Finland and the five hospital catchment areas included here for all the vaccination strategies. For these scenarios, the basic reproduction number $R_{\text{eff}} = 1.0$ and the mobility value $\tau = 0.0$.

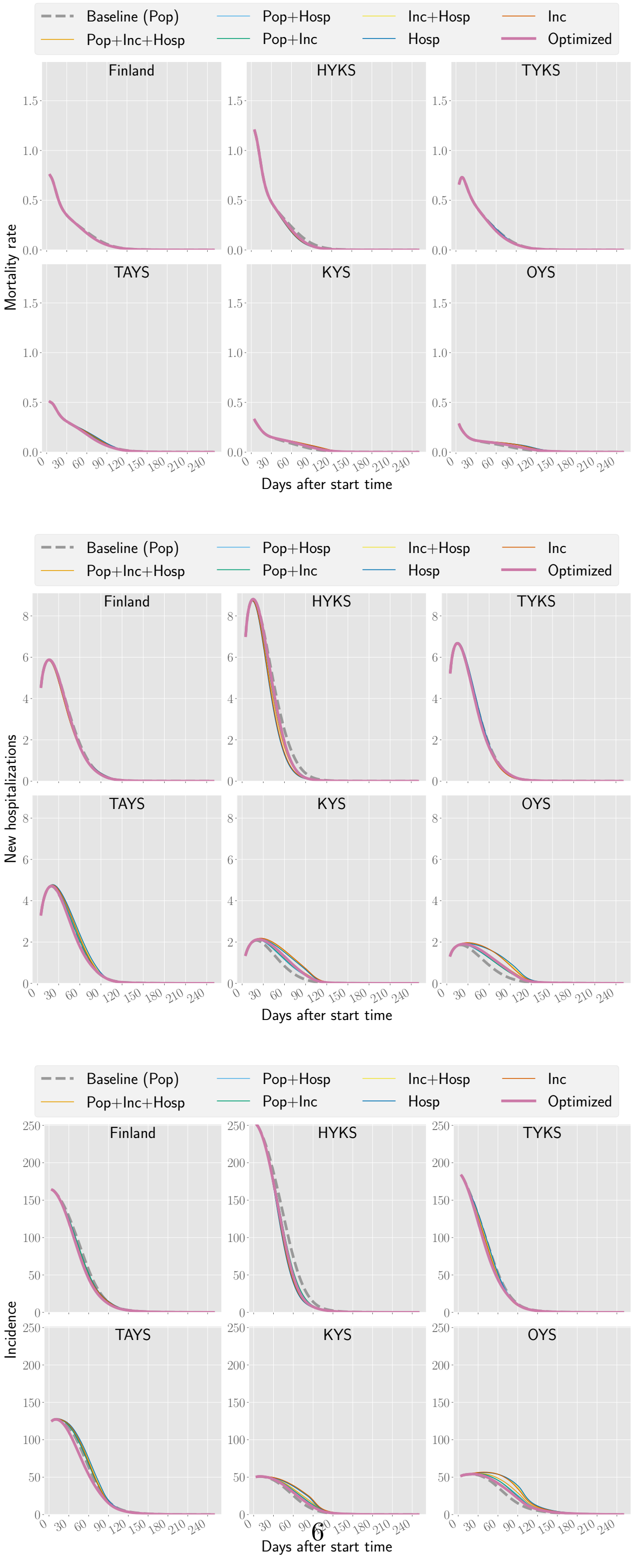

Fig. E: Different metrics per million inhabitants in Finland and the five hospital catchment areas included here for all the vaccination strategies. For these scenarios, the basic reproduction number $R_{\text{eff}} = 1.0$ and the mobility value $\tau = 0.5$.

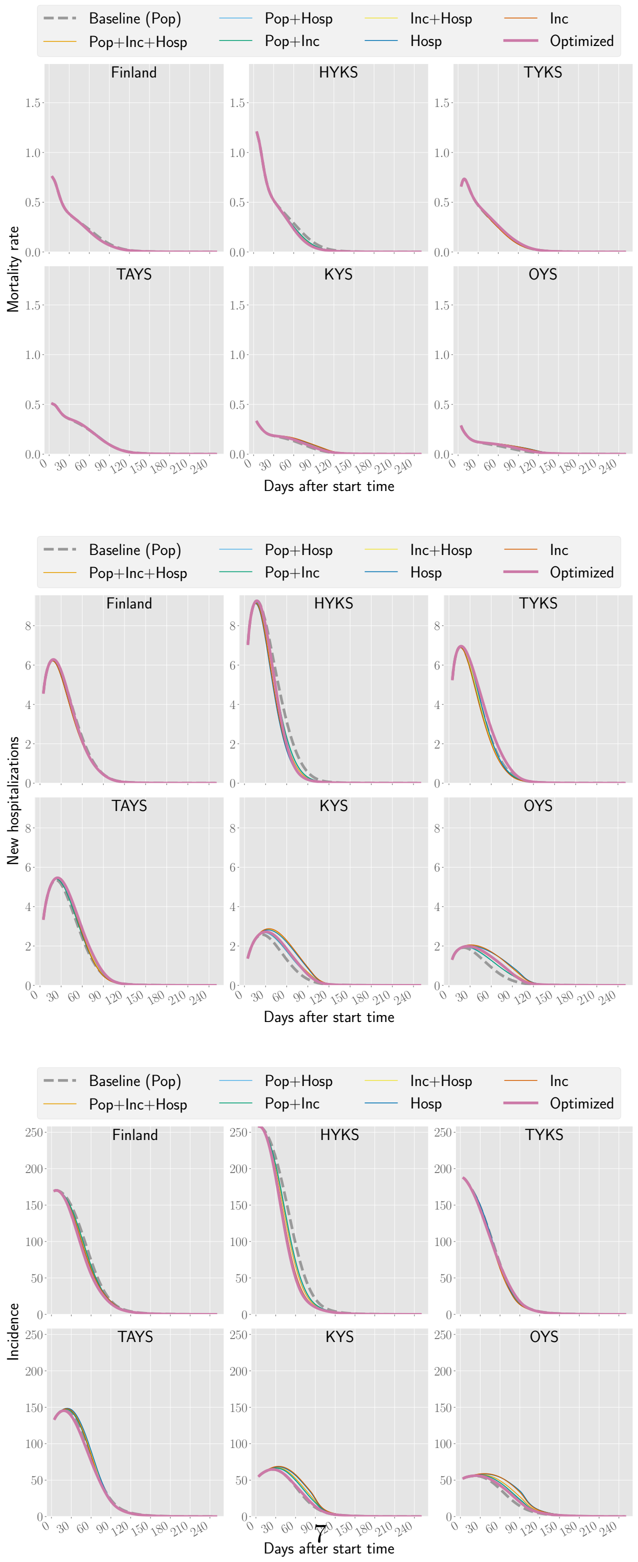

Fig. F: Different metrics per million inhabitants in Finland and the five hospital catchment areas included here for all the vaccination strategies. For these scenarios, the basic reproduction number $R_{\text{eff}} = 1.0$ and the mobility value $\tau = 1.0$.

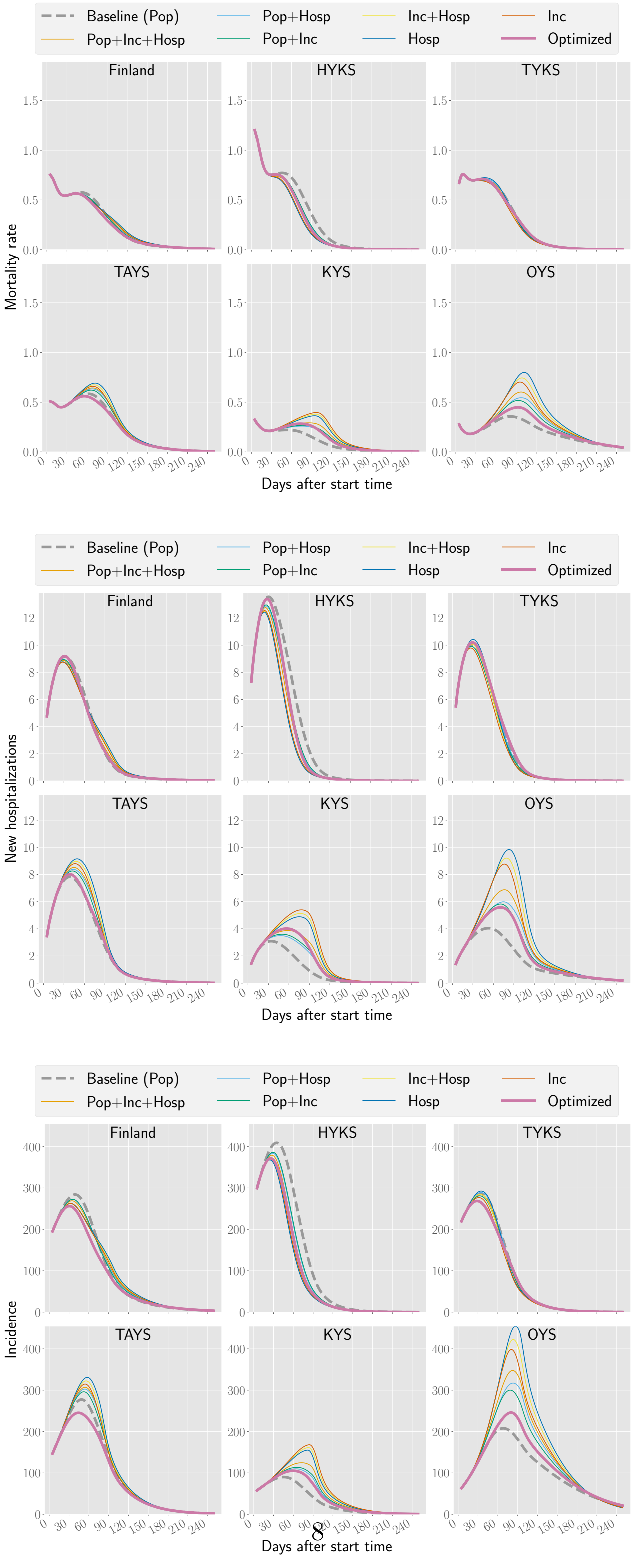

Fig. G: Different metrics per million inhabitants in Finland and the five hospital catchment areas included here for all the vaccination strategies. For these scenarios, the basic reproduction number $R_{\text{eff}} = 1.25$ and the mobility value $\tau = 0.0$.

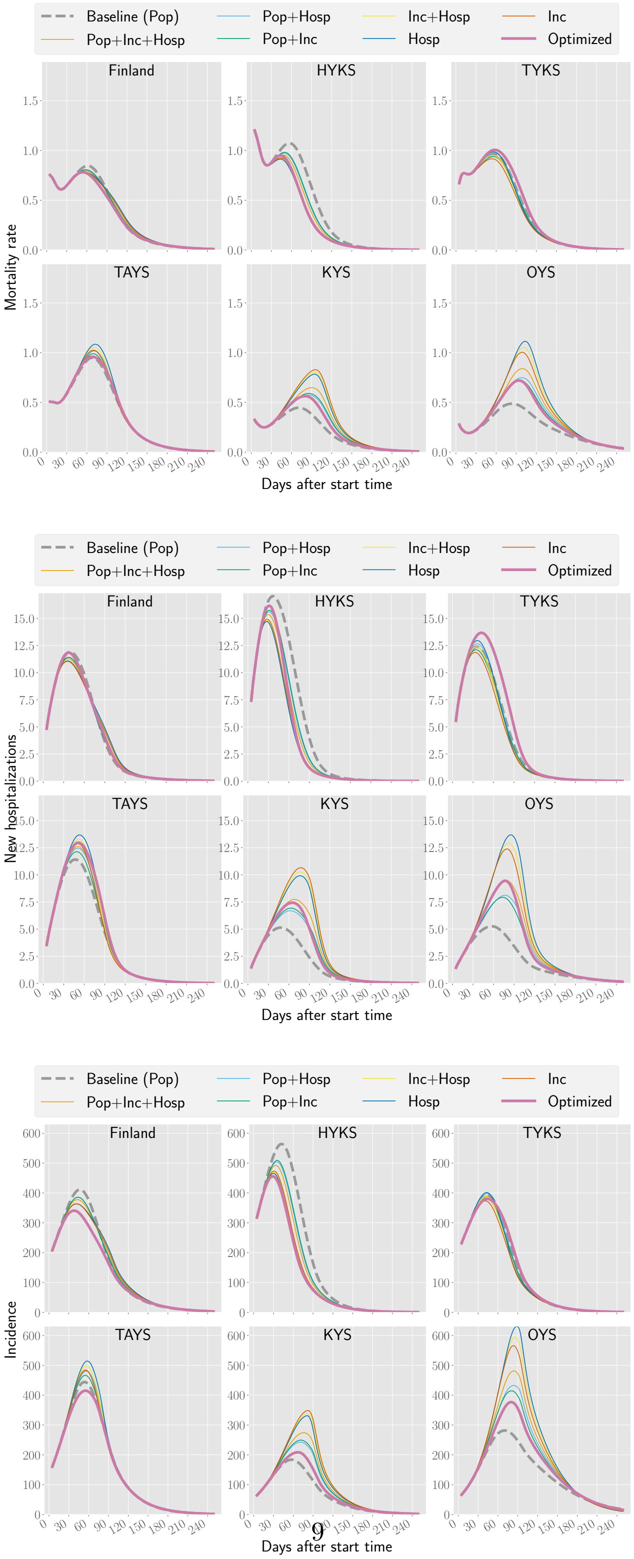

Fig. H: Different metrics per million inhabitants in Finland and the five hospital catchment areas included here for all the vaccination strategies. For these scenarios, the basic reproduction number $R_{\text{eff}} = 1.25$ and the mobility value $\tau = 0.5$.

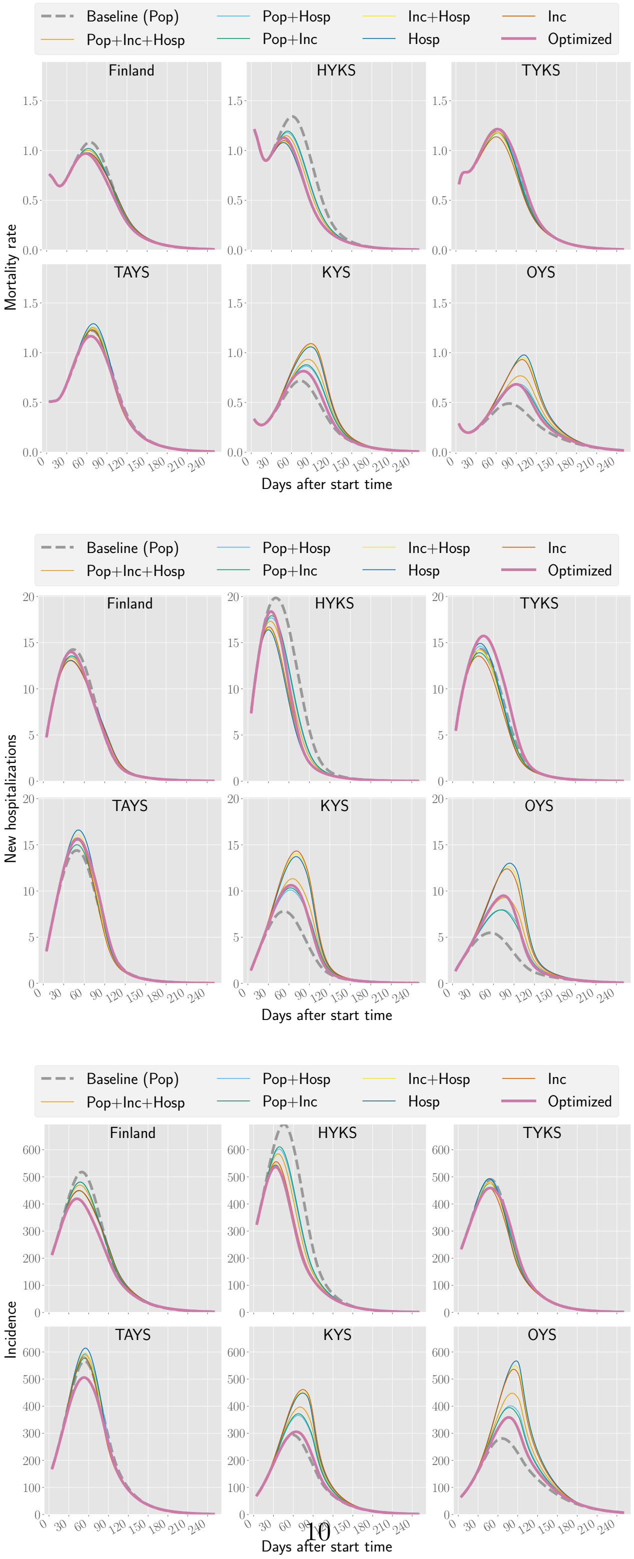

Fig. I: Different metrics per million inhabitants in Finland and the five hospital catchment areas included here for all the vaccination strategies. For these scenarios, the basic reproduction number $R_{\text{eff}} = 1.25$ and the mobility value $\tau = 1.0$.

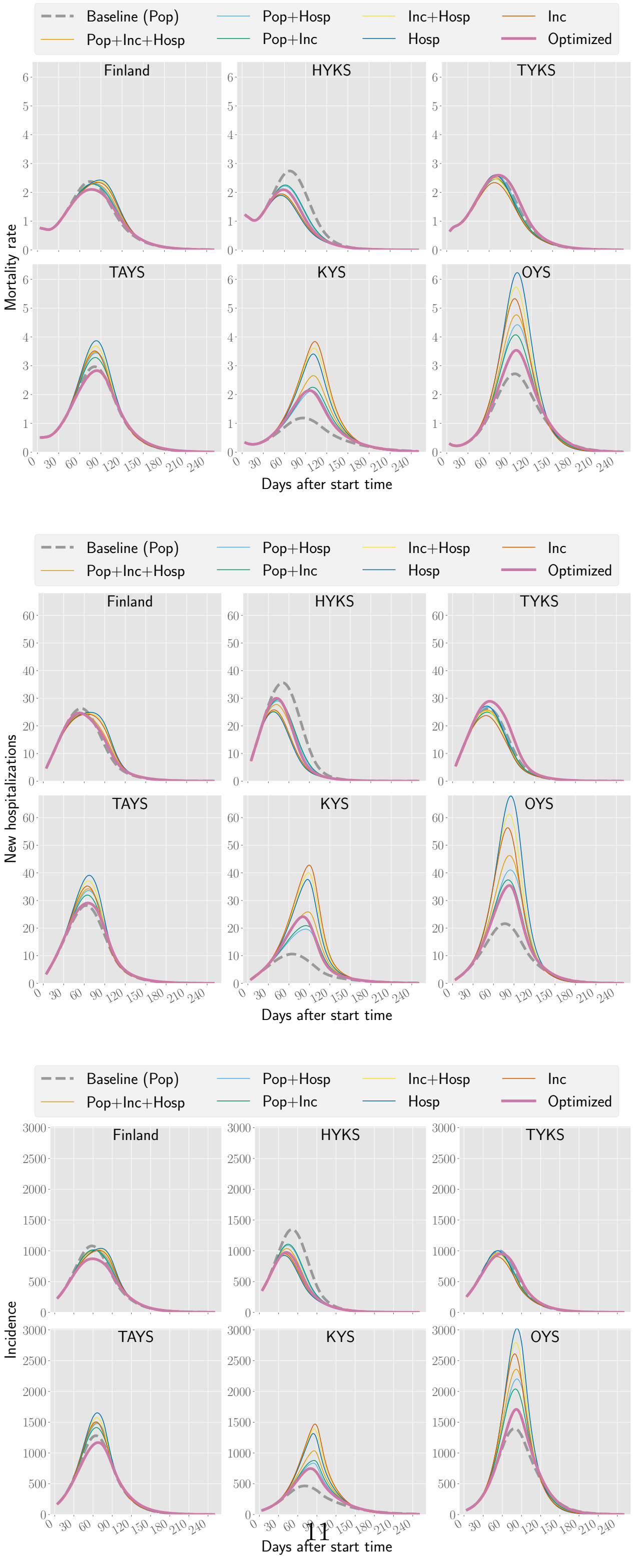

Fig. J: Different metrics per million inhabitants in Finland and the five hospital catchment areas included here for all the vaccination strategies. For these scenarios, the basic reproduction number $R_{\text{eff}} = 1.5$ and the mobility value $\tau = 0.0$.

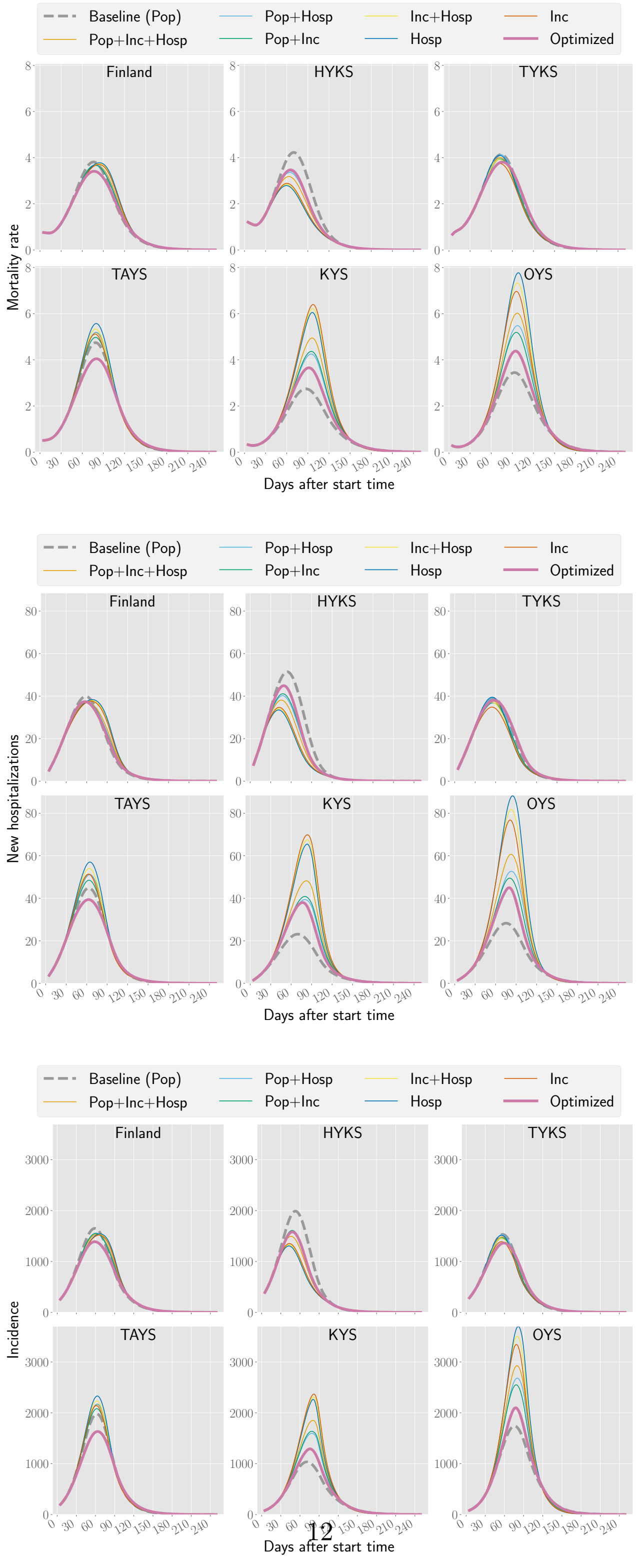

Fig. K: Different metrics per million inhabitants in Finland and the five hospital catchment areas included here for all the vaccination strategies. For these scenarios, the basic reproduction number $R_{\text{eff}} = 1.5$ and the mobility value $\tau = 0.5$.

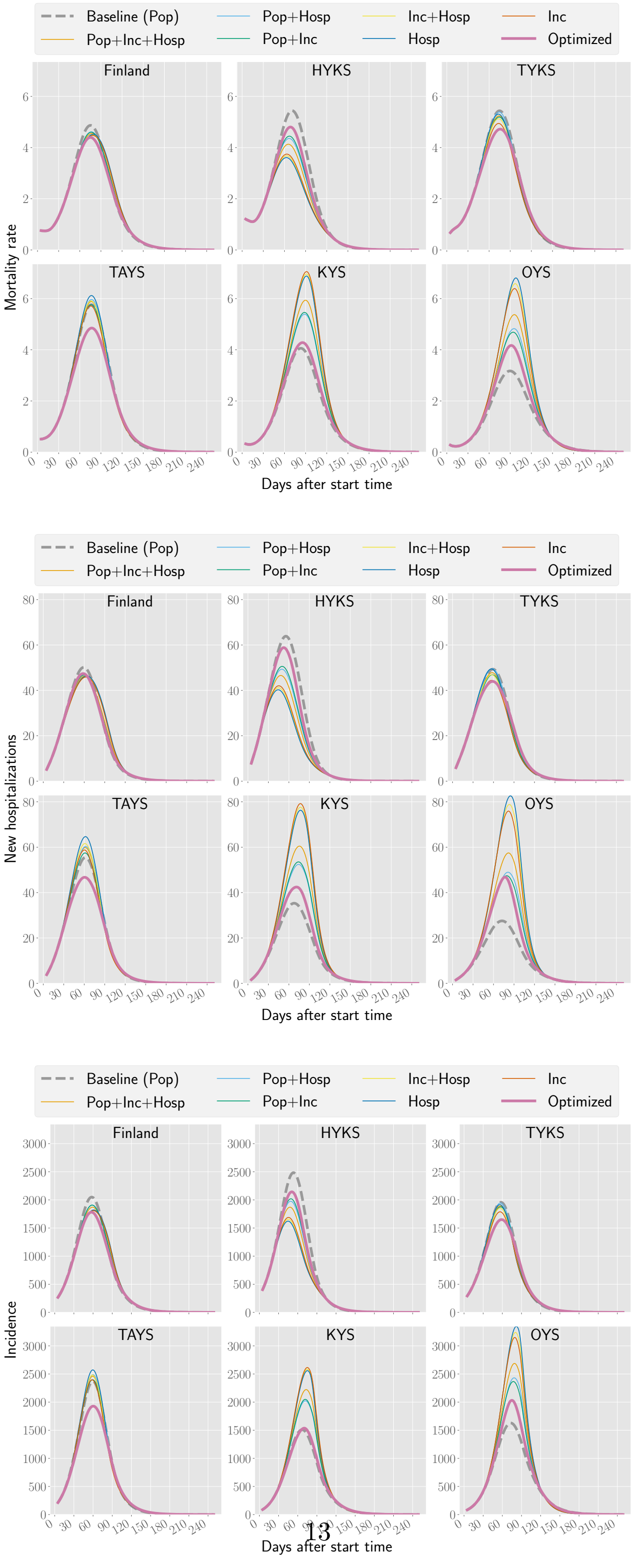

Fig. L: Different metrics per million inhabitants in Finland and the five hospital catchment areas included here for all the vaccination strategies. For these scenarios, the basic reproduction number $R_{\mathrm{eff}} = 1.5$ and the mobility value $\tau = 1.0$.

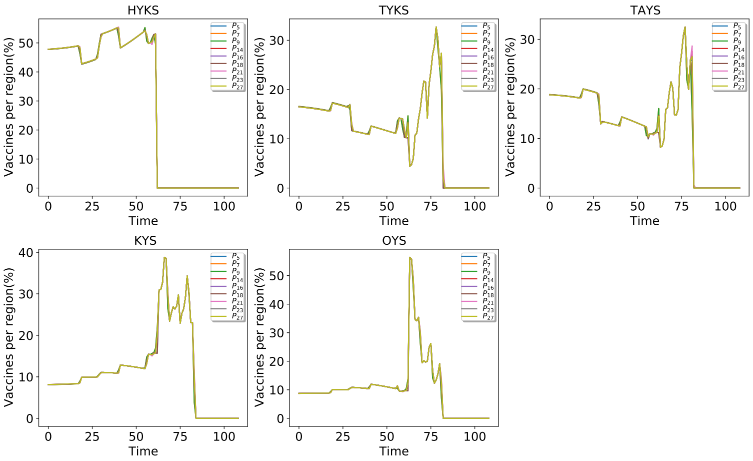

Fig. M: Percentage of vaccine doses allocated by the optimized strategy to regions for different values of e, ω and π.

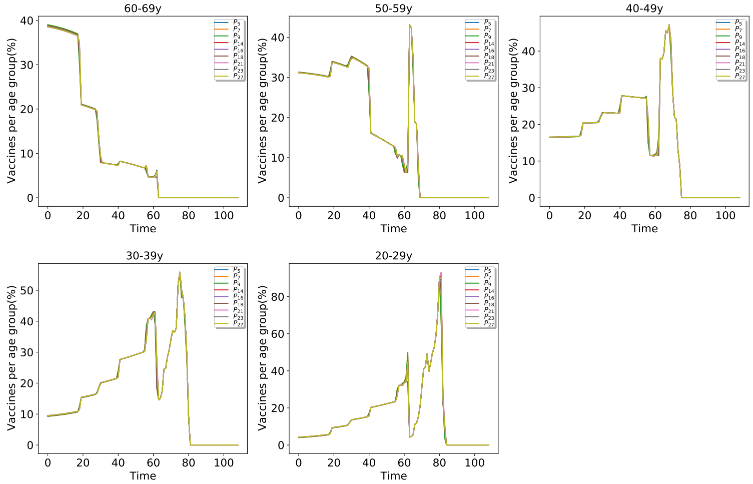

Fig. N: Percentage of vaccine doses allocated by the optimized strategy to age groups for different values of e, ω and π. Each $P_i, i \in \{5, 7, 9, 14, 16, 18, 21, 23, 27\}$ represents a different combination of e, ω and π taken from (3.1) in S1 Appendix.

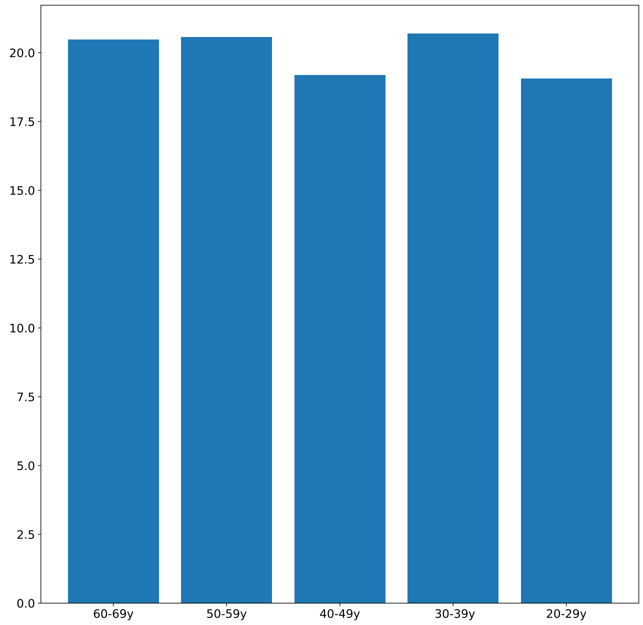

Fig. O: Percentage of vaccines which each age group would receive proportionally to the age group size.
